# Cross-modulation of pathogen-specific pathways enhances malnutrition during enteric co-infection with *Giardia lamblia* and enteroaggregative *Escherichia coli*

**DOI:** 10.1371/journal.ppat.1006471

**Published:** 2017-07-27

**Authors:** Luther A. Bartelt, David T. Bolick, Jordi Mayneris-Perxachs, Glynis L. Kolling, Gregory L. Medlock, Edna I. Zaenker, Jeffery Donowitz, Rose Viguna Thomas-Beckett, Allison Rogala, Ian M. Carroll, Steven M. Singer, Jason Papin, Jonathan R. Swann, Richard L. Guerrant

**Affiliations:** 1 Division of Infectious Diseases, Department of Medicine, University of North Carolina at Chapel Hill, Chapel Hill, North Carolina, United States of America; 2 Center for Gastrointestinal Biology and Disease, Department of Medicine, University of North Carolina at Chapel Hill, Chapel Hill, North Carolina, United States of America; 3 Division of Infectious Diseases and International Health, Department of Medicine, University of Virginia, Charlottesville, Virginia, United States of America; 4 Division of Computational and Systems Medicine, Department of Surgery and Cancer, Imperial College London, United Kingdom; 5 Department of Biomedical Engineering, University of Virginia, Charlottesville, Virginia, United States of America; 6 Division of Pediatric Infectious Diseases, Children’s Hospital of Richmond at Virginia Commonwealth University, Richmond, Virginia, United States of America; 7 Department of Biology, Georgetown University, Washington, DC, United States of America; New York University, UNITED STATES

## Abstract

Diverse enteropathogen exposures associate with childhood malnutrition. To elucidate mechanistic pathways whereby enteric microbes interact during malnutrition, we used protein deficiency in mice to develop a new model of co-enteropathogen enteropathy. Focusing on common enteropathogens in malnourished children, *Giardia lamblia* and enteroaggregative *Escherichia coli* (EAEC), we provide new insights into intersecting pathogen-specific mechanisms that enhance malnutrition. We show for the first time that during protein malnutrition, the intestinal microbiota permits persistent *Giardia* colonization and simultaneously contributes to growth impairment. Despite signals of intestinal injury, such as IL1α, *Giardia*-infected mice lack pro-inflammatory intestinal responses, similar to endemic pediatric *Giardia* infections. Rather, *Giardia* perturbs microbial host co-metabolites of proteolysis during growth impairment, whereas host nicotinamide utilization adaptations that correspond with growth recovery increase. EAEC promotes intestinal inflammation and markers of myeloid cell activation. During co-infection, intestinal inflammatory signaling and cellular recruitment responses to EAEC are preserved together with a *Giardia*-mediated diminishment in myeloid cell activation. Conversely, EAEC extinguishes markers of host energy expenditure regulatory responses to *Giardia*, as host metabolic adaptations appear exhausted. Integrating immunologic and metabolic profiles during co-pathogen infection and malnutrition, we develop a working mechanistic model of how cumulative diet-induced and pathogen-triggered microbial perturbations result in an increasingly wasted host.

## Author summary

Malnourished children are exposed to multiple sequential, and oftentimes, persistent enteropathogens. Intestinal microbial disruption and inflammation are known to contribute to the pathogenesis of malnutrition, but how co-pathogens interact with each other, with the resident microbiota, or with the host to alter these pathways is unknown. Using a new model of enteric co-infection with *Giardia lamblia* and enteroaggregative *Escherichia coli* in mice fed a protein deficient diet, we identify host growth and intestinal immune responses that are differentially mediated by pathogen-microbe interactions, including parasite-mediated changes in intestinal microbial host co-metabolism, and altered immune responses during co-infection. Our data model how early life cumulative enteropathogen exposures progressively disrupt intestinal immunity and host metabolism during crucial developmental periods. Furthermore, studies in this co-infection model reveal new insights into environmental and microbial determinants of pathogenicity for presently common, but poorly understood enteropathogens like *Giardia lamblia*, that may not conform to existing paradigms of microbial pathogenesis based on single pathogen-designed models.

## Introduction

Childhood malnutrition and its resultant host developmental, metabolic, and immunologic sequelae continue to affect 156 million children less than five years of age worldwide [1]. Impaired child growth attainment is epidemiologically associated with 1) alterations in resident intestinal microbiota (dysbiosis) [1, 2]; 2) increased susceptibility to multiple concurrent and recurrent enteric pathogens [3, 4]; 3) intestinal dysfunction together with markers of increased intestinal myeloid [5] and T-cell activation (termed Environmental Enteropathy (EE))[3, 6]; and 4) perturbations in gut microbial-host co-metabolism [7]. Emerging data from the Malnutrition and Enteric Diseases (Mal-ED) multisite international study has revealed that cumulative pathogen exposures confer a high associated risk for poor growth [8]. These exposures are diverse, with prokaryotic pathogens enteroaggregative *Escherichia coli* and the oftentimes persistent protozoan *Giardia lamblia* among the most commonly detected [9, 10]. Elucidating mechanistic pathways by which these diverse microbial triggers interact to potentiate the malnourished condition could improve restorative interventions for malnourished children. Indeed, data from randomized control therapeutic trials expose knowledge gaps in our biological understanding of microbial drivers of malnutrition [11–15]. Inconsistent or only partial benefits are achieved from interventions that target a single component of this pathogenesis such as nutrient supplementation alone [11] or in combination with broad-spectrum antibacterials [12, 13], anti-parasitic drugs [14], or anti-inflammatory agents [15]. Furthermore, field studies of endemic pediatric *Giardia* have associated *Giardia* with decreased risk of severe diarrhea and inflammatory biomarkers of EE, yet increased risk for growth impairment, suggesting that for some pathogens, novel pathways may contribute to impaired child development [9].

Using murine models of malnutrition that result in diet-dependent changes in the microbiota [16] we have published that challenge with the human EAEC isolate (strain 042) [17] or *G*. *lamblia* (assemblage B, strain H3, cysts) [18] is sufficient to impair growth and disrupt mucosal architecture, but with unique intestinal pathologies. We have not previously investigated whether and how these individual pathogens interact with the resident microbiota or with one another, in part due to the use of antimicrobial mediated microbiota depletion to support human pathogen colonization in mice [17–21]. Also, although xenotransplantation of feces from discordant healthy and malnourished children into gnotobiotic murine recipients demonstrate the functional ability of human-derived microbial communities to selectively recapitulate phenotypes of undernutrition, dysbiosis, [1, 2, 22], and disrupted metabolism [1, 23], these studies are only beginning to examine the influence of enteropathogenic bacteria accompanying the dysbiosis [22, 23] and have yet to uncouple these effects from direct or residual influences of intestinal eukaryotes also present in donor feces [1]. Thus, while existing murine models provide insight into individual nutritional and microbial triggers that influence gut function, metabolism, and host growth, none to date have intentionally examined the integrated effects of dysbiosis with sequential and diverse pathogen exposures common in malnourished children.

To address how gut microbial adaptations to undernutrition combine with cumulative enteropathogen burdens to influence host growth, mucosal immune responses, and metabolism, we developed a new integrated model of protein-malnutrition induced microbial disruption and multi-pathogen enteropathy. *G*. *lamblia* and EAEC, pathogens commonly detected in malnourished children, were selected as pathogens of interest. In addition to identifying new pathogen-specific pathways that contribute to malnutrition, we demonstrate co-modulation of mucosal immune and metabolic responses that converge to worsen host growth. Furthermore, gut microbial-mediated proteolysis was amplified in the increasingly wasted host along with exhaustion of co-metabolic adaptations in energy regulatory and compensatory metabolic pathways.

## Results

### *Giardia* overcomes microbiota-mediated pathogen clearance during protein malnutrition, and combines with protein malnutrition to promote growth impairment, small intestinal 16S abundance, and altered mucosal immunity

**Fig 1:**
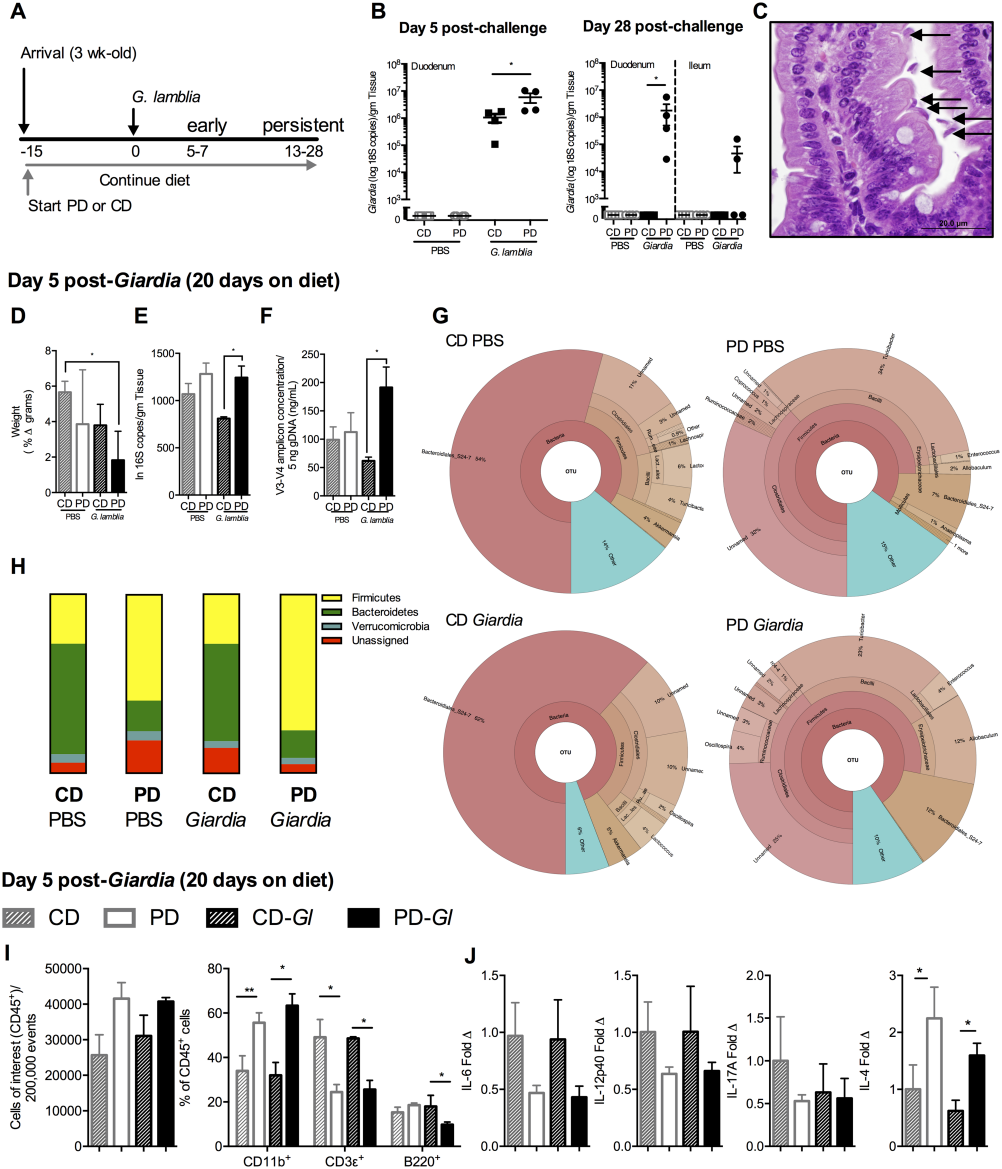
Protein malnutrition in weaned mice disrupts *Giardia* clearance, promotes increased microbial-pathogen abundance in small intestine, and establishes cytokine profiles associated with persistent *Giardia* infection. (A) Experimental timeline of *Giardia* challenge in 3 week-old C56Bl/6 mice initiated on either control diet (CD) or protein-deficient diet (PD) upon arrival and continued throughout the duration of the experiment. *G*. *lamblia* (Assemblage B, H3) infection occurred on experimental day 0. Data for this and subsequent experiments were obtained during either early timepoints when mice on both diets remained infected with *Giardia* (day 5–7) or later timepoints (up to 28 days) (persistent) when only mice fed PD diet remained infected. (B) Duodenal *Giardia* burden at day 5 (left) and duodenal and ileal *Giardia* burden on day 28 (right) post-challenge in either CD or PD-fed mice and uninfected controls. (C) Histopathology of duodenum in infected mice fed PD diet (20x, H&E, arrows designate trophozoites). (D) Growth in mice (as % weight increase between day 5 and day 0). (E) Small intestinal bacterial abundance by total 16S rRNA universal primers and (F) quantification of the V3-V4 region amplification product. (G) Krona visualizations of 16S rRNA OTUs in the upper small intestine high-abundance taxa (>10,000 reads per OTU) in mice fed CD (left) or PD (right) 5 days after *Giardia lamblia* (bottom) compared with age and diet-matched controls (top) as indicated. (H) Phyla-level 16S rRNA relative abundances in upper small intestine 5 days after *G*. *lamblia* challenge compared with age and diet-matched controls. (I) Flow cytometry of duodenal lamina propria leukocytes (LPL) day 5 post-challenge, stained for leukocytes (CD45^+^) and frequency of myeloid (CD11b^+^), T- (CD3^+^) and B- (B220^+^) cells and (J) Cytokine protein levels in duodenum shown as fold change relative to uninfected CD-fed controls (PBS). For all studies n = 4/group; *P<0.05, **P<0.01.

Murine intestinal microbiota can differentially prevent prolonged *Giardia lamblia* colonization, even in T and B-cell deficient hosts. Thus, we and other investigators have used continuous antibiotics (ampicillin, vancomycin, neomycin) (Abx) in drinking water to enhance *G*. *lamblia* infection [18–21]. Using this Abx cocktail we previously published that *G*. *lamblia* (Assemblage B, strain H3 cysts) challenge results in detectable shedding at 10^4^−10^5^/gram feces by qPCR of the 18S small ribosomal subunit through the first 5–7 days post-challenge (early infection). But unlike clearance following *G*. *lamblia* (Assemblage A, strain WB trophozoites) challenge, *G*. *lamblia* H3 shedding increases by ~ 2 logs after day 9 and remains consistent through 4–6 weeks together with small intestinal trophozoite colonization (persistent infection) [18]. To test the hypothesis that the disrupted intestinal 16S community during protein malnutrition [16] would functionally impair microbiota-mediated colonization resistance [24], we eliminated Abx from the model. Weaned mice were fed either a protein deficient (2% protein) diet (PD) or an isocaloric but protein sufficient (20% protein) control diet (CD) for 15 days prior to challenge with 10^6^ *G*. *lamblia* H3 (Assemblage B) cysts (Fig 1A). We previously published that this duration of acclimation on diet is sufficient to establish discrepant 16S rRNA genetic profiles [16]. Small intestinal tissues harvested at 5 days (early) and 28 days (persistent) post-infection (dpi) demonstrated higher abundance of *Giardia* on day 5 in mice fed PD and only mice fed PD remained infected through 28 days by qPCR (Fig 1B). Histopathology confirmed the presence of mucosal-associated *Giardia* trophozoites in H3 cyst-challenged mice fed PD (Fig 1C). In separate experiments, we confirmed that parasites persisted in mice fed CD and challenged with 10^6^ *G*. *lamblia* H3 cysts if concurrently treated with Abx. *Giardia* was detected in the duodenum of abx-treated mice fed CD at 35 dpi, and regardless of diet, *Giardia* was detected in stools through 42 dpi (S1 Fig). Finally, consistent with the greater infectious potential of the partially stomach-acid resistant parasite cyst stage compared with the excysted trophozoite stage, we confirmed that regardless of Abx, only H3 cysts and not axenized H3 trophozoites were sufficient to achieve consistent *Giardia* colonization by both light microscopy and qPCR (S1 Fig) in this model.

Similar to what we observed previously in abx-treated mice [18], protein deficiency combined with *Giardia* to impair host growth (P<0.05 PD-*Giardia* vs uninfected CD-fed control in Fig 1D). *Giardia* infection in mice fed a PD diet also had greater duodenal bacterial abundance, measured by both universal 16S rRNA qPCR/gram tissue and V3-V4 specific amplicon product, than infected mice fed CD (P<0.05) (Fig 1E and 1F). Consistent with several features of microbial alterations in both malnourished children [25] and protein-deficient diet fed mice [26], the duodenal 16S rRNA composition in mice fed PD demonstrated an increased Firmicutes:Bacteroidetes ratio (S1 Fig) that was mainly driven by an increase in the abundance of Clostridiales (Fig 1G). *Giardia* tended to enhance this skew towards Firmicutes together with a reduction in Bacteroidetes from 12%—7% (Fig 1H). Thus, rather than excluding *Giardia*, the small intestinal microbiota in mice fed the protein deficient diet permitted persistent *Giardia* infection whereas abx were necessary for prolonged parasite detection in mice fed the CD diet.

To examine whether PD in this model had interfered with protective mucosal responses against *Giardia* we performed flow cytometry on upper small intestinal lamina propria in the same mice. Regardless of infection, mice fed PD demonstrated a skew toward increased myeloid cells (CD11b^+^) with reciprocal reductions in T-cells (CD3ε^+^) among CD45^+^ cells analyzed compared with mice fed the CD-diet (Fig 1I). In addition, mice fed PD demonstrated a reduction in B-cell frequency (B220^+^) at 5 dpi compared with infected mice fed CD (P<0.05) (Fig 1I). Concurrently, we analyzed mucosal production of key cytokines that promote *Giardia* clearance, such as IL-6 and IL-17A [27, 28], compared with those that resembled the profile of prolonged infections in children (increased IL-4) [28] (Fig 1J). Corresponding to persistent infection, mice fed PD demonstrated a trend toward decreased IL-6, IL-17A, and IL12p40, with a significant increase in IL-4 (P<0.05 for IL-4), irrespective of *Giardia* infection.

### Intestinal microbiota determine growth outcomes during giardiasis

**Fig 2:**
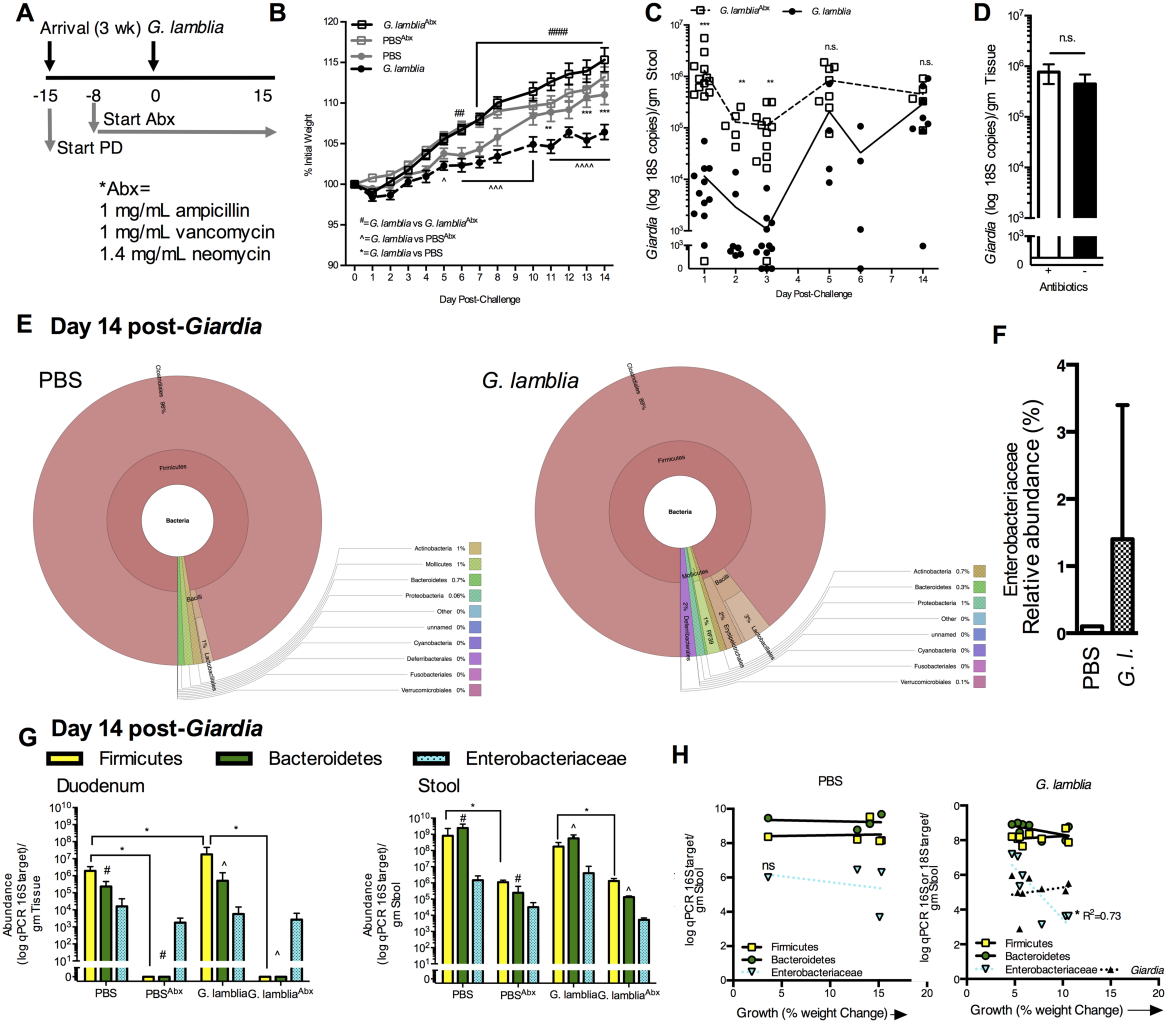
*Giardia* interacts with resident microbiota to impair host growth during protein malnutrition. (A) Experimental timeline. 3-week-old C57Bl/6 mice were initiated on the PD diet upon arrival. Antibiotic (Abx)-treated animals received antibiotics vancomycin, neomycin, and ampicillin continuously *ad libitum* in drinking water beginning 8 days prior to *G*. *lamblia* challenge and throughout the duration of the experiment. (B) Growth curves of each experimental group as percent of initial weight beginning on the day of *G*. *lamblia* challenge (Day 0). **P<0.01, *** P<0.001 (*G*. *lamblia* vs. PBS); ^P<0.05, ^^^P<0.01, ^^^^P<0.001 (*G*. *lamblia* vs. PBS^Abx^), and ^##^ P<0.01 and ^####^ P<0.0001 (*G*. *lamblia* vs. *G*.*lamblia*^Abx^) (n = 11-13/group in combined replicate experiments). (C) Effect of continuous antibiotics on serial *Giardia* fecal shedding and (D) day 14 duodenum burden as determined by 18S qPCR. (E) Krona visualization of fecal 16S V3-V4 OTUs in PBS control (left) and *G*. *lamblia* infected (right) mice at 14 days post-challenge. (F) Fecal Enterobacteriaceae abundance by 16S V3-V4 OTUs in PBS and *G*. *lamblia* (*G*.*l*.) infected mice at 14 days post-challenge. (G) Abundance of Firmicutes, Bacteroidetes and *Enterobacteriaceae* by qPCR in duodenum (left) and feces (right) day 14 after *G*.*lamblia* challenge (*P<0.05, ****P<0.0001), ^#^P<0.05, ^^^P<0.05 as indicated (n = 4 = 8/group). (H) Correlation between growth as % weight change at 14 days after G. lamblia challenge (such that 0 = no change in weight and 15 = 15% weight increase) and fecal qPCR abundance of designated bacterial taxa or *G*. *lamblia* in uninfected (left) and *G*. *lamblia* challenged mice (right).

Having established that *Giardia* incorporates into a disrupted intestinal microbiota in mice fed the PD diet, we next investigated the role of resident microbiota as determinants of growth impairment during *Giardia* infection in PD diet fed mice (Fig 2A). Continuous exposure to the antibacterials that have no anti-giardial activity (Abx) prevented *Giardia*-induced growth impairment (Fig 2B) even despite an early increase in *Giardia* fecal shedding (Fig 2C) and a similar intestinal *Giardia* burden through 14 dpi (Fig 2D). The Abx exposure resulted in a fecal dominance of *Lactococcus* (>98%) regardless of infection (Fig 1S). In non-Abx treated mice, there were no significant differences in fecal 16S rRNA composition between infected and non-infected mice (Fig 2E), although *Enterobacteriaceae* tended to be over-represented in *Giardia* infected mice (Fig 2F). Targeted qPCR to determine absolute abundance of predominant taxa (Firmicutes and Bacteroidetes) as well as *Enterobacteriaceae* identified reductions in both Firmicutes and Bacteroidetes to below the limit of detection in the duodenum and 3–4 log decreases in the feces during Abx treatment regardless of infection (Fig 2G).

In non-Abx treated *Giardia*-infected mice there was a 1.5 log increase in Firmicutes (P<0.05) in the duodenum (Fig 2G). In addition, consistent with findings that increased numbers of *E*. *coli* in small intestinal aspirates recovered from patients with giardiasis correlate with greater symptom severity [29], increased fecal *Enterobacteriaceae* abundance at 15 dpi in non-Abx treated mice was a better predictor of poor growth in individual *Giardia-*infected mice than *Giardia* burden in either stool or duodenum (Fig 2H).

### *Giardia* combines with EAEC to worsen malnutrition and both pathogens contribute to altered mucosal immune responses during co-infection

**Fig 3:**
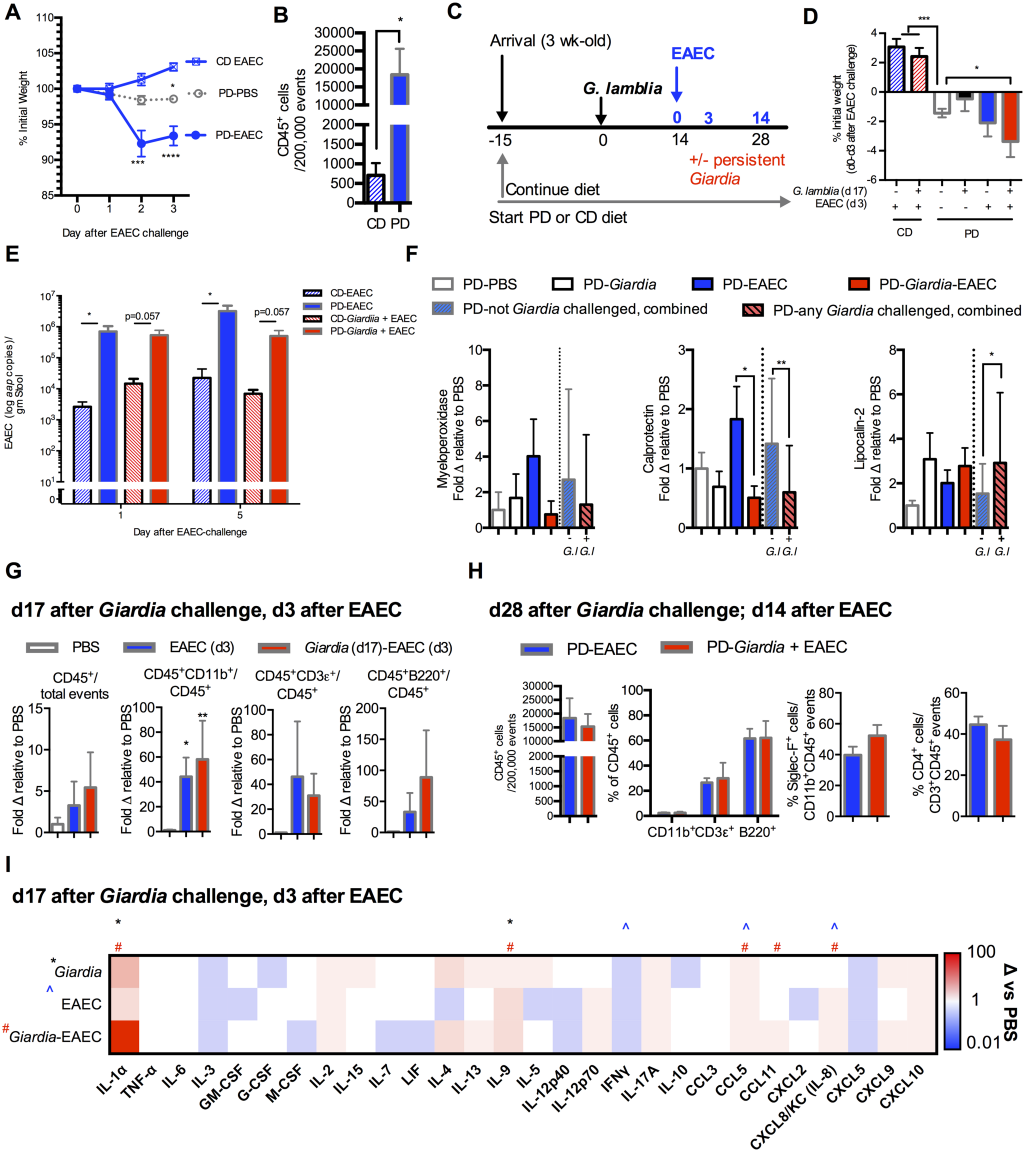
*Giardia* and EAEC combine to worsen growth during protein malnutrition and persistent *Giardia* infection alters intestinal immune responses to EAEC. (A) Impact of 10^9^ EAEC042 challenge on growth (% initial weight) of PD-fed and CD-fed mice compared with PD-fed uninfected controls (Day 0 = 28 days on diet) (n = 4/group; *P<0.05 PD-PBS vs CD-EAEC day 3; ***P<0.001 and ****P<0.0001 PD-EAEC vs PD-PBS day 2 and day 3). (B) Flow cytometry of ileum lamina propria leukocytes (CD45+) through 14 days post-EAEC infection (n = 4/group; *P<0.05). (C) Experimental timeline for persistent *G*. *lamblia*-EAEC co-infection challenge model. 3-week-old C57Bl/6 mice were initiated on either the PD or the CD diet upon arrival and for 15 days prior to *G*. *lamblia* challenge. Sequential challenge with EAEC occurred during persistent (d 14) *G*. *lamblia* infection. (D) Impact of prior *Giardia* exposure on growth following EAEC co-infection. Depicted is growth through 3 days after EAEC co-infection (32 days on diet, 17 days after *Giardia* infection) (n = 4 per group (CD diet groups) and 6-17/group (PD diet groups); ***P<0.001 PD uninfected vs. either CD-EAEC or CD-*G*.*lamblia*-EAEC; *P<0.05 PD-*G*. *lamblia*-EAEC vs. PD uninfected) (E) EAEC shedding on day 1 and day 5 post-challenge in CD or PD-fed mice either with or without prior *Giardia* exposure as indicated. (n = 4/group; *P<0.05 as indicated). (F) Luminal inflammatory biomarkers (Myeloperoxidase (MPO), Calprotectin, and Lipocalin-2) depicted as fold change relative to PBS uninfected controls 3 days after EAEC and 13 days after *G*. *lamblia* challenge (n = 7-13/group). Data to the right of the dashed line in all groups represents differences dichotomized to whether mice received *G*. *lamblia* challenge (any *Giardia* challenged, combined) or not (not *Giardia* challenged, combined) (n = 12-24/group). Data for fecal MPO also includes two individual combined experiments, at 13–17 days after *Giardia* and 3–7 days after EAEC (n = 7-13/group). (G) Flow cytometry of ileum lamina propria leukocytes (CD45+), and proportions of myeloid (CD11b+), T- (CD3+) and B- (B220+) cells 3 days after EAEC042 challenge (17 days after *G*. *lamblia* challenge) in PD-fed mice as indicated (n = 4-8/group; *P<0.05 (EAEC vs PBS), **P<0.01 (*Giardia*-EAEC vs PBS). Data is shown as fold change relative to uninfected PBS controls. (H) Flow cytometry of ileum lamina propria (left) leukocytes (CD45^+^), (middle) proportion of myeloid (CD11b^+^), T- (CD3^+^) and B- (B220^+^) and (right) eosinophils (Siglec-F^+^CD11b^+^) and CD4^+^ T-cells at 14 days after EAEC042 challenge (28 days after *G*. *lamblia* challenge) in PD-fed mice as indicated. (I) Intestinal protein levels 3 days after EAEC042 challenge (17 days after *G*. *lamblia)* in PD-fed mice. 29 of Luminex 32-target panel results shown in heatmap. Data depicted as fold change relative to uninfected controls (range 0.01 to 100). (*P<0.05 for *Giardia*, ^P<0.05 for EAEC, and #P<0.05 for *Giardia*-EAEC vs uninfected controls, n = 6-12/group).

To test whether alterations in intestinal microbiota and mucosal immune responses during persistent *Giardia* infection would enhance or diminish growth impairment during enteropathogen co-infection, we next developed a sequential co-infection model using one of the most common pathogen isolated in malnourished children, EAEC [10]. For these experiments we used EAEC042 that elicits acute myeloid cell inflammation during other nutrient deficient states [17]. First, we established that challenge with 10^9^ EAEC042 in mice fed the PD diet but not mice fed CD diet led to rapid weight loss (7% body weight compared with uninfected PD-fed controls (P<0.001 3 dpi) (Fig 3A) and mucosal inflammation that persisted through 14 days post-EAEC challenge (Fig 3B). Next, we acclimated mice on either the PD or CD diet for 15 days prior to *Giardia* exposure and then sequentially challenged with EAEC042 during the persistent phase (14 dpi) of *Giardia* infection (Fig 3C). The two pathogens combined to enhance weight loss in mice fed the PD diet (~100% greater loss of initial weight, *P*<0.05 in co-infected mice compared with uninfected PD-fed controls) (Fig 3D). In mice fed CD and co-infected with both pathogens, no weight loss was observed (Fig 3D). *Giardia* did not influence EAEC042 stool shedding which was 2 logs greater in mice fed the PD diet as determined by qPCR of the EAEC-specific *aap* target (Fig 3E).

*Giardia*, however, did alter inflammatory markers of environmental enteropathy when present alone and during EAEC co-infection in protein deficient fed mice. Myeloperoxidase (MPO) a product of activated neutrophils, was variably detected in the mice fed protein deficient. Fecal MPO tended to be elevated in response to either pathogen alone, but paradoxically decreased to levels similar to uninfected controls in co-infected mice (Fig 3F). Calprotectin (Cp), another marker of myeloid cell activation, was elevated only in EAEC042 mono-infected animals, but was decreased in any *Giardia* infected group (Fig 3F). Lipocalin-2 (LCN), a marker of either neutrophil or epithelial cell activation was elevated only in *Giardia-*infected mice regardless of EAEC co-infection.

Immune responses in the mucosal compartment were also altered in persistent *Giardia* infected mice later challenged with EAEC. EAEC infection led to significant increases in myeloid lineage (CD11b^+^ cells) in the ileum at 17 dpi *Giardia* challenge and 3 dpi EAEC challenge (Fig 3G). The increased proportion of lymphocytes (both CD3ε^+^ and B220^+^ cells) at 28 dpi *Giardia* challenge and 14 dpi EAEC challenge (Fig 3H) was similar in either EAEC mono-infected or co-infected mice. In contrast, total LPLs, particularly lymphocytes (CD3ε+ and B220+ cells) were decreased during persistent (17 dpi) *Giardia* infection compared with uninfected controls (S2 Fig). Using a broad-based luminex 32-plex panel we performed an unbiased analysis of cytokine and chemokine responses on all protein deficient diet fed mice at 17 dpi *Giardia* challenge and 3 dpi EAEC challenge. We detected 28 of 32 targets in at least 2 mice in each group that are shown as fold change relative to uninfected controls (Fig 3I). Both pathogens modulated the cytokine/chemokine response alone and during co-infection. In all conditions, IL1α, a pro-inflammatory alarmin that is released by enterocytes during intestinal injury [30], was elevated in all groups and reached significance in *Giardia* infected mice (~30-fold) and robustly increased in co-infection (~80-fold). IL-9 was significantly elevated in *Giardia* mono-infected and by ~20-fold in co-infected mice, together with a tendency towards greater IL-4 and IL-13. Each group demonstrated a decrease in IFNγ, that was significant in EAEC mono-infected mice. CCL5 was elevated (~2 fold) in EAEC infected and co-infected mice. Consistent with the early expansion of myeloid cells in EAEC infected mice, CXCL8 (IL-8/KC) was also elevated in EAEC and co-infected mice (~1.6 fold). CCL11 (eotaxin) was uniquely elevated (~6-fold) only in co-infected mice. This change corresponded to a trend toward increased eosinophils (CD45^+^SiglecF^+^) among myeloid cells in co-infected compared with EAEC mono-infected mice (52% vs 39%, ns) at later timepoints (Fig 3H). Changes in select cytokines and chemokines in *Giardia* mono-infected mice from early (5 dpi) to persistent (17 dpi) timepoints compared with uninfected controls revealed alterations in mucosal immune responses during persistent *Giardia* infection. *Giardia* lead to progressive increases in IL-1α (P<0.05) and IL-2 (P<0.05) as well as IL-4 and IL-13, but IFNγ progressively decreased (P<0.05) in persistently infected mice (S2 Fig).

### Co-infection enhances gut microbial host proteolysis and abolishes energy regulatory compensatory responses during malnutrition

**Fig 4:**
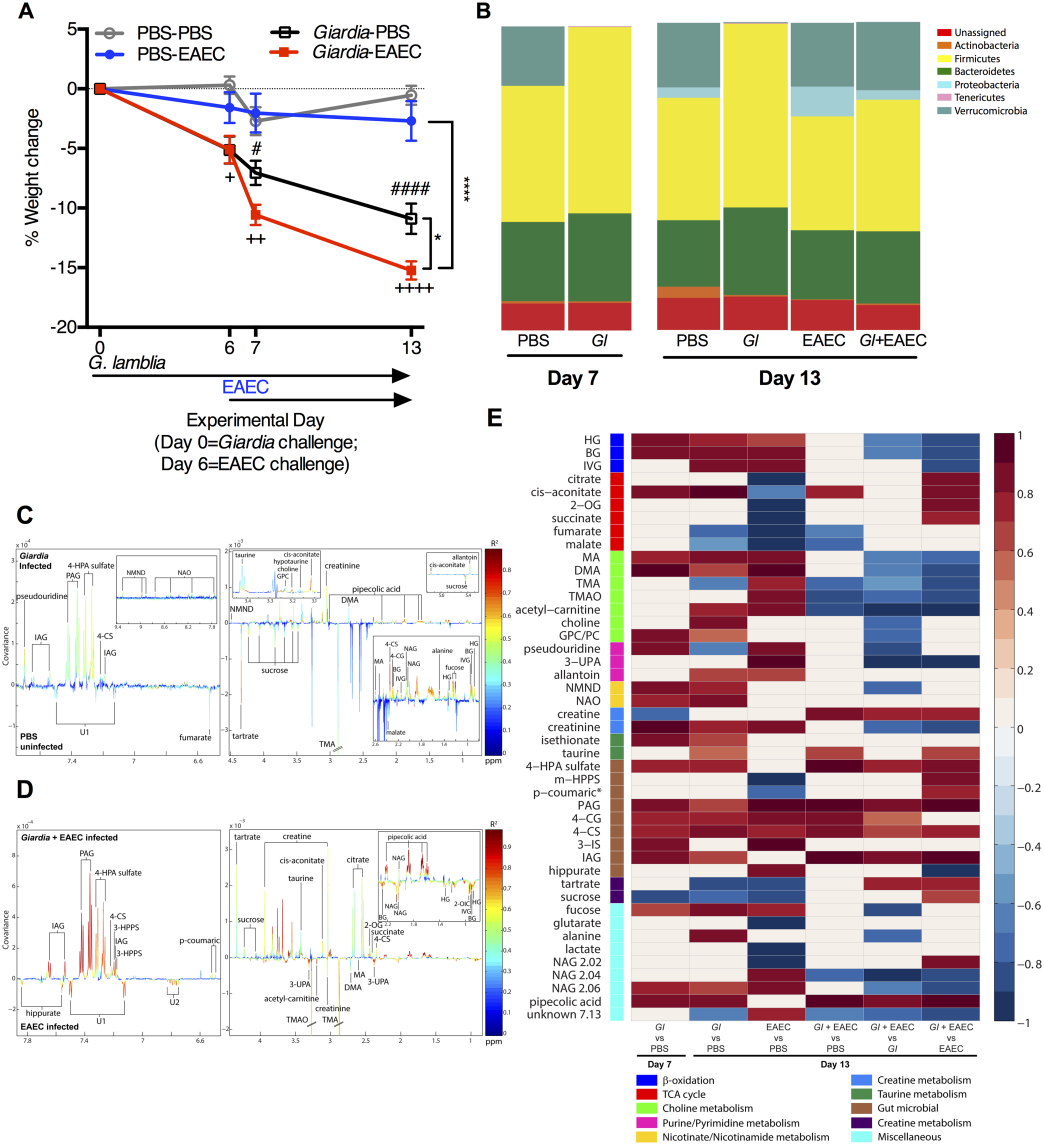
EAEC combines with *Giardia* to enhance microbial host co-metabolic perturbations and co-infection exhausts regulatory host energy expenditure adaptations during malnutrition. C57Bl/6 males were initiated on the protein deficient diet upon arrival at 3 weeks of age and then challenged with *G*. *lamblia* 7 days later. Six days after *G*. *lamblia*, two groups were challenged with enteroaggregative *E*. *coli* (EAEC). Uninfected controls received PBS-PBS sham gavages at each infection timepoint. (n = 6/group). (A) Depicted is growth as percentage of starting weight beginning on the day of *G*. *lamblia* infection (Experimental day 0 (Day 0)) and through 13 days after *Giardia* challenge (7 days after EAEC challenge) (Day 13). (^+^*P*<0.05, ^++^*P*<0.01, ^++++^*P*<0.001 *Giardia*-PBS vs PBS-PBS; ^#^*P*<0.05, ^####^*P*<0.001 *Giardia*-PBS vs PBS-EAEC; *P<0.05 *Giardia*-EAEC vs. *Giardia*-PBS Day 7 and Day 13; ****P<0.0001 *Giardia*-EAEC vs. PBS-EAEC Day 7 and Day 13). (B) Phylum-level relative operational taxonomic unit (OTU) abundances determined after amplification and sequencing of the V3-V4 region of 16S rRNA gene in fecal samples from uninfected, *Giardia*-infected, EAEC-infected, and co-infected mice on experimental days 7 and 13 as indicated. (C,D) OPLS-DA correlation coefficient plots indicating the variation between (C) *Giardia* infected (Day 13) and uninfected PBS controls (Day 13) (Q^2^Y = 0.40; P = 0.02) or (D) co-infection (Day 13) versus EAEC mono-infection (Day 13) (Q^2^Y = 0.82; p = 0.001) (n = 4-5/group). Correlation coefficients plots were generated with the use of a back-scaling transformation to display the contribution of each metabolite to the sample classification. Positive peaks indicate metabolites that were excreted in greater amounts in the *Giardia* infected (C) or co-infected (D) mice, and negative peaks indicate metabolites that were excreted in lower amounts. The color scale represents the significance of the correlation for each metabolite to the class membership with red indicating stronger significance and blue indicating weaker significance. (E) Heat map of significant changes in urinary metabolites measured using 1H NMR spectroscopy associated with OPLS-DA models. Represented as correlation coefficients (R) (red = increased; blue = decreased) in *G*. *lamblia* (*Gl*) infection at Day 7 and Day 13 versus age-matched uninfected (PBS) controls, EAEC mono-infection Day 13 versus age-matched uninfected (PBS) controls, or co-infection (*Gl*+EAEC) Day 13 compared with age-matched uninfected controls (PBS) or either mono-infection alone. Abbreviations: 2-OG, 2-oxoglutarate; 3-IS, 3-indoxylsulfate; 3-UPA, 3-ureidopropionic acid; 4-HPA, 4-hydroxyphenylacete; 4-CG, 4-cresolglucuronide; 4-CS 4-cresyl sulfate; DMA, dimethylamine; BG, butyrylglycine; GPC, α-glycerophosphocholine; HG, hexanoylglycine; IAG, indole acetyl gulconate; IVG, isovalarylglycine; MA, methylamine; m-HPPS, m-hydroxyphenylpropionylsulfate; NAG, N-acetyl glutamine; NAO, nicotinamide-N-oxide; NMND, N-methyl- nicotinamide; PAG, N-phenylacetylglycine; TMA, trimethylamine; TMAO, trimethylamine-N-oxide; UK, unknown.

To determine whether either pathogen alone or the pathogens in combination altered gut microbial host metabolism, we performed 16S rRNA sequencing in feces simultaneously with urinary metabolic profiling (metabonomics) using ^1^H nuclear magnetic resonance (NMR) spectroscopy in mice fed the PD diet (Fig 4). In this experiment, weaned mice were highly susceptible to weight loss following 10^6^ *G*. *lamblia* H3 cyst challenge, that was further potentiated following EAEC co-infection six days later (Fig 4A). Focusing first on 16S rRNA sequencing, in *Giardia* mono-infected mice, phyla-level changes at day 7 and day 13 showed consistent relative increases in Firmicutes and reductions in Verrucomicrobia (*Akkermansia mucinophila*) in *Giardia* mono-infected mice (Fig 4B). Anaerobes such as Clostridiales members (day 7 and day 13 after *G*. *lamblia* challenge) and *Turicibacter* (day 7 after *G*. *lamblia* challenge) as well as *Enterococcus sp*. (day 13 after *Giardia* challenge) accounted for the Firmicutes expansion in *Giardia* mono-infected mice (S3 Fig). Either *Giardia* or EAEC mono-infected mice had a reduction in *Bifidobacterium pseudolongum*. In EAEC mono-infected or co-infected mice *Enterobacteriaceae* were increased relative to uninfected controls or *Giardia* mono-infection (S3 Fig). Phyla-level 16S rRNA composition in co-infected mice otherwise more closely resembled EAEC mono-infection.

Orthogonal projection to latent structures-discriminant analysis (OPLS-DA) coefficient plots identified a range of urinary metabolic perturbations induced by *Giardia* infection on both day 7 and day 13 (Fig 4C) (Q^2^Y = 0.40; P = 0.02 vs uninfected PBS controls) many of which were also elevated in co-infected compared with EAEC mono-infected mice (Q^2^Y = 0.82; p = 0.001) (Fig 4D). We observed no significant difference in the OPLS-DA metabolic profiles of *Giardia* infected mice between day 7 or 13 days post-challenge (Q^2^Y = 0.33 R^2^X = 0.24, P = 0.12). Significantly altered metabolites are summarized in a heat map in Fig 4E along with their correlation to class membership. Focusing first on metabolites unique to *Giardia* infection (Fig 4E), consistent with *Giardia* trophozoite reliance upon on host-derived lipids for membrane synthesis and optimal growth (ie. lecithin, gylcocholic and taurocholic bile) [31], *Giardia*-infected mice demonstrated increased excretion of bile acid constituents, phosphatidylcholine (PC) coupled with choline breakdown metabolites methylamine (MA) and dimethylamine (DMA) and the taurine metabolite isethionate. These indicators of bile acid deconjugation and lipid breakdown were present on both day 7 and day 13 post-*Giardia* challenge (Fig 4E). Increases in MA and DMA occurred independent of a concurrent increase in the microbial-dependent precursor trimethylamine (TMA) or its hepatic oxidized metabolite TMAO, a biochemical pattern resembling that observed with Kwashiorkor-type malnutrition [1], and were thus suggestive of increased choline availability in the small intestine rather than downstream gut microbial-dependent choline breakdown. Alanine, a by-product of *Giardia* glucose fermentation, was elevated at day 13 post-*Giardia* challenge in mono-infected mice, while pipecolic acid, one of the most abundant amino acid byproducts of *Giardia* metabolism *in vitro* [32] was identified in *Giardia-*infected mice at both timepoints, regardless of co-infection. *Giardia* also enhanced gut microbial-host co-metabolites of aromatic amino acids including tyrosine (4-cresol glucuronide (4-CG) and 4-cresyl sulfate (4-CS) and 4-hydroxyphenylacetyl (4-HPA) sulfate), tryptophan (3-indoxyl sulfate (3-IS) and indole-3-acetylglycine (IAG)), and phenylalanine (phenylacetylglycine (PAG)). Increases in urinary β-oxidation metabolites, accumulation of the early tricarboxylic acid cycle intermediate *cis*-aconitate, and changes in muscle metabolites creatine and creatinine indicated altered host energy utilization in *Giardia*-infected mice. In addition, methylated nicotinamide derivatives capable of regulating energy expenditure (*N*-methylnicotinamide (NMND) and nicotinamide-N-oxide (NAO)) [23] were increased in *Giardia* infected mice. Consistent with the finding that increased urinary NMND predicts catch-up growth in undernourished children [7], persistently *Giardia-*infected mice fed the protein deficient diet developed ‘overshoot’ growth gains compared with uninfected age and diet-matched controls upon re-nourishment (switched from the PD to the CD diet on 42 dpi) (S3 Fig).

The *Giardia-*induced changes in gut microbial host co-metabolites of proteolysis either persisted (4-HPA sulfate, IAG) or overlapped (PAG, 4-CG, 4-CS) with those seen during EAEC infection alone, and these metabolites were even further magnified in co-infected mice (Fig 4E). However, EAEC-mediated increases in TMA and TMAO (Fig 4E), indicative of microbial-dependent choline breakdown, were reversed in *Giardia* co-infected mice (Fig 4E), and resembled the metabolic perturbation in choline metabolism of *Giardia* infection alone (Fig 4E). Similarly, elevated taurine excretion in *Giardia-* infected mice persisted through co-infection (Fig 4E). Whereas either infection increased lipid oxidation evident in increased β-oxidation breakdown products (hexanoylglycine, butyrylglycine, and isovalarylglycine) along with the β-oxidation pathway precursor acetyl-carnitine, metabolism during co-infection shifted away from β-oxidation as indicated by a decrease in acetyl-carnitine and downstream β-oxidation metabolites. Concurrently, co-infection led to an inversion of creatine:creatinine ratios, suggesting altered muscle metabolism compared with either infection alone (Fig 4E). Finally, host energy expenditure adaptations via the nicotinamide pathway (NMND and NAO) during *Giardia* infection alone (Fig 4E) were extinguished following EAEC co-infection (Fig 4E).

## Discussion

**Fig 5:**
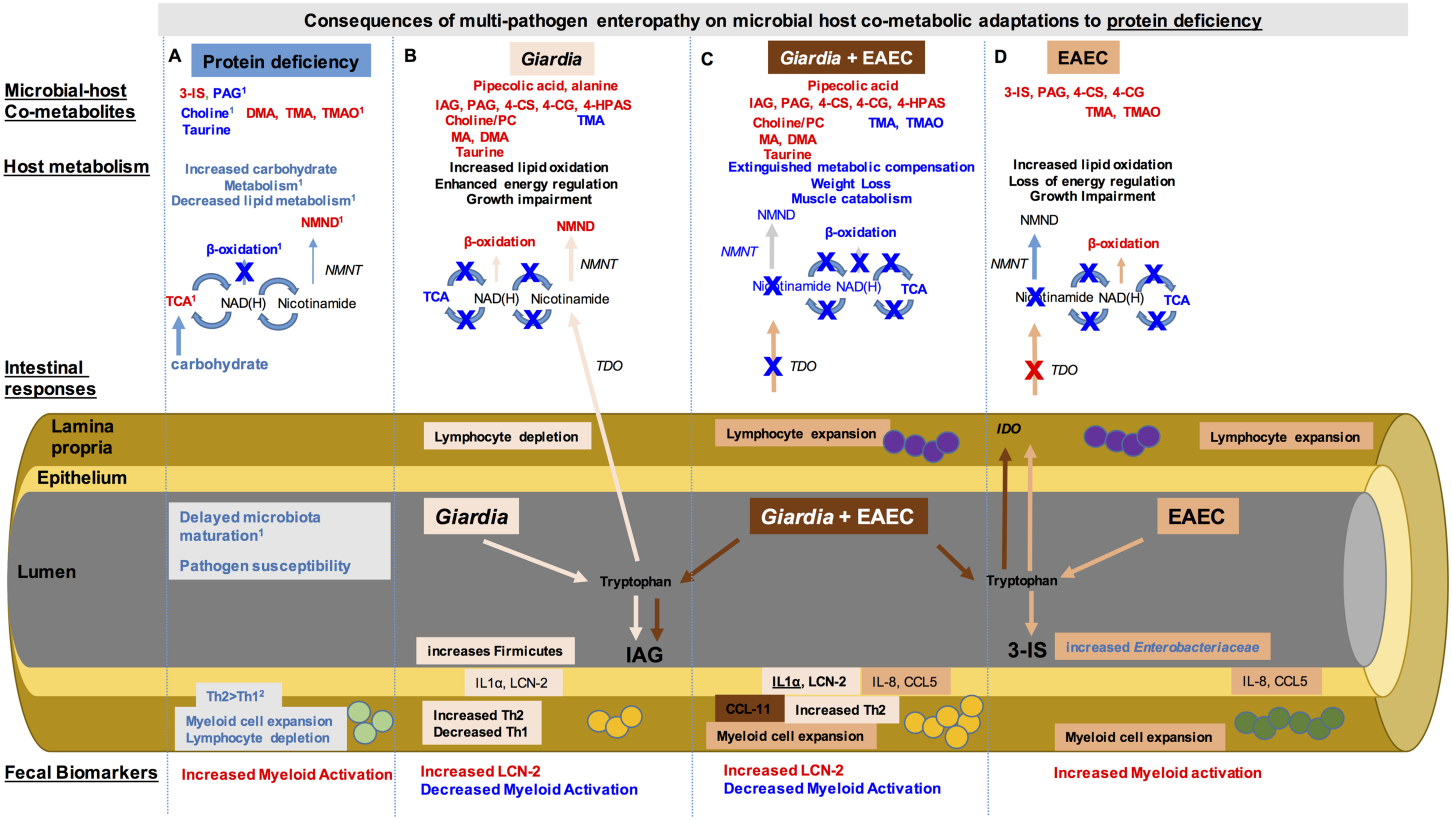
Co-enteropathogens co-modulate host immune and metabolic responses during protein deficiency that converge to worsen malnutrition. Integrated studies identified that protein deficiency (Column A) results in an increase (red) in microbial host co-metabolites of tryptophan (3-indoxyl sulfate, 3-IS) as well as choline (dimethylamine, DMA; trimethylamine, TMA; and trimethylamine oxide, TMAO) whereas endogenous choline and taurine excretion were decreased (blue). Host metabolism indicated increased carbohydrate metabolism through the tricarboxyclic acid cycle (TCA) and decreased β-oxidation metabolites. Methylation of nicotinamide was increased leading to increased N-methyl nicotinamide (NMND). The intestinal microbiota during protein deficiency are ‘immature’ and result in increased pathogen susceptibility. This microbiota restructuring is accompanied by proportionally increased myeloid cells in the lamina propria, but decreased lymphocytes and a Th2>Th1 skew. During *Giardia* infection (light orange) (Column B), there are unique metabolites (such as pipecolic acid and alanine). Additional increases in metabolites of tryptophan (indole-3-acetylglycine), phenylalanine (phenylacetylglycine, PAG; 4-hydroxyphenylacetyl (4-HPA) sulfate), and tyrosine (4-cresyl sulfate, 4-CS, and 4-cresolglucuronide 4-CG) indicate further microbial-mediated proteolysis, whereas increased choline, posphatidylcholine (PC), MA and DMA suggest increased choline absorption and decreased TMA indicates limited microbial choline breakdown. These metabolic changes occur despite little perturbation in the intestinal microbial composition. Host metabolism through TCA is shutdown, limiting production of NAD(H), however, NMND excretion is further increased, suggesting generation of nicotinamide through alternative pathways such as tryptophan 2,3 dioxygenase (TDO). *Giardia* further depletes lymphocytes in the intestinal mucosa while enhancing the Th2>Th1 skew, despite signals of intestinal injury IL1α and lipocalin-2 (LCN-2). EAEC infection (dark orange) (Column D) similarly enhances microbial proteolytic metabolites, though tryptophan is broken down to 3-IS rather than IAG. Increased intestinal microbial choline metabolism leads to increases in TMA and TMAO, opposite to *Giardia* infection. Although host TCA metabolism is diminished as occurs during *Giardia* infection, NMND is not increased. Rather, cellular expansion of lymphocytes in response to EAEC and the broadly acting chemokine CCL5 limits alternative nicotinamide generation pathways through the actions of indoleamine 2,3 dioxygenase (IDO). EAEC also enhances myeloid cell recruitment with concurrent increases in IL-8. During co-infection (rust) (Column C) the microbial host co-metabolites resemble those occurring during *Giardia* infection alone, however, host metabolic compensatory responses are lost. Unique metabolic products of *Giardia* metabolism, such as pipecolic acid, are detectable. Inflammatory cell recruitment resembles EAEC infection, but with a *Giardia*-mediated altered immune response profile, with the unique elevation of CCL11. Fecal markers of myeloid activation that are diminished during *Giardia* infection are also attenuated during co-infection, implicating either parasite- or metabolite-induced changes in myeloid cell phenotypes. ^1^Mayneris-Perxachs, et al. 2016 [16] ^2^ Bartelt, et al. 2015 [35].

Multiple and diverse pathogen exposures are hypothesized to cause intestinal dysfunction, also termed Environmental Enteropathy (EE), in malnourished children. In the present study, we modeled co-infection with two of the most commonly isolated pathogens in malnourished children, *Giardia lamblia* and enteroaggregative *Escherichia coli* (EAEC). We used protein deficiency in weaned mice to investigate how microbial-specific pathways intersect to impair host growth, mucosal inflammation, and metabolism during malnutrition. Our integrated nutritional, microbial, immunological, and metabolic observations add insight into how changes in resident microbiota combine with cumulative enteropathogen exposures to interfere with host growth and metabolic adaptations to protein malnutrition. For the first time we demonstrate that a resident microbiota that is permissive to enteropathogen colonization also simultaneously promotes growth impairment during persistent *Giardia* infection. Although *Giardia* was insufficient to induce intestinal inflammation characteristic of EE-like changes, despite evidence of mucosal injury (IL1α), the parasite had a profound effect on gut microbial-host co-metabolism. EAEC, on the other hand, promoted robust expansion of lamina propria cells coupled with secretion of myeloid (CXCL8 (IL-8)) and lymphoid (CCL5) chemokines. Together, these pathogens synergistically increased signals of intestinal injury, IL1α, and CCL11. EAEC-dependent increases in myeloid cells were preserved in co-infected mice; however, persistent *Giardia* infection resulted in diminished myeloid cell specific activation markers (Cp and to a lesser degree MPO) consistent with parasite-mediated alterations in host immune pro-inflammatory responses. Strikingly, these non-invasive co-pathogens resulted in an increasingly proteolytic microbiota that dominated the co-metabolic profile (specifically leading to increased tryptophan, tyrosine, and phenylalanine co-metabolites), despite relatively restricted changes in the 16S rRNA composition. Simultaneously, host metabolic adaptation to protein deficiency progressively declined, eventually resulting in the loss of host-mediated nicotinamide-pathway energy regulation, and disrupting lipid oxidation up-regulation and muscle metabolism in the increasingly malnourished host. A working model of these specific pathogen-mediated microbial, immunologic, and metabolic alterations are shown in Fig 5.

Our findings support that an ability to better compete for restricted resources in the intestinal environment is one mechanism whereby enteropathogens may more successfully infect malnourished hosts. For example, the protein deficient diet contains 0.34% rather than the 3.4% arginine contained in the 20% protein sufficient control diet [16]. In weaned mice, this protein deficient diet recapitulates several dysbiotic features described in malnourished children: altered maturation of the fecal intestinal microbiota [16, 33], increased susceptibility to *Giardia* and EAEC, and increased microbial-mediated tryptophan breakdown [7, 16]. In the present study we also observed an altered Firmicutes:Bacteroidetes ratio in the protein deficient diet-fed mice [25], that was modestly increased at early timepoints after *Giardia* infection. In contrast arginine-supplementation has been shown to increase the abundance of Bacteroidetes relative to Firmicutes in the small intestine [34]. Since *Giardia* is a microaerophilic protozoan that can utilize either glucose or arginine for growth and replication, we speculate that a diet-dependent decrease in Bacteroidetes reduced bacterial competition for arginine. A limitation of 1H-NMR profiling is an inability to directly detect arginine metabolites (ornithine, citrulline), and thus we could not determine whether *Giardia* infection was sufficient to further magnify host arginine deficiency. However, consistent with *Giardia* use of arginine in order to evade host immune defenses through arginine-deiminase (ADI) [35], the continued decline in IFNγ in mice infected with *Giardia* could be a result of the actions of *Giardia* ADI to skew dendritic cell TLR-responses away from pro-inflammatory cytokines [36]. Furthermore, a reduction in B-cells as seen in *Giardia* infected mice, is similar to other models of arginine deficiency [37]. Also, unlike arginine-mediated increases in Bacteroidetes that can enhance TLR-dependent mucosal immune responses [38] similar to some specific *Lactobacilli* that facilitate *Giardia* clearance in mice [34], the protein deficient diet led to decreases in pro-inflammatory cytokines associated with *Giardia* [27, 28, 39, 40] or EAEC [41] clearance: namely IL-6, IL-17A, and IFNγ. Rather, reciprocal increases in IL-4, a correlate of prolonged duration of *Giardia* shedding in children [18, 42] were seen. Interestingly, like the arrested maturation of the microbiota during protein deficient conditions [16], this relative shift toward a predominately Th2-type cytokine mileu also resembles that of the neonatal period [43]. This Th2-type cytokine shift can also be differentially induced via upregulation of thymic stromal lymphopoietin (TSLP) in response to Firmicutes-rich altered Schaedler flora [44]. These collective findings suggest that the isolated protein deficiency in this model establishes a threshold nutrient deficiency that is sufficient to disrupt microbiota-mediated pathogen exclusion, and adds insights into why postnatal *Giardia* acquisition (up to 6 months of age) may be such a vulnerable period for longitudinal growth impairment [7, 45].

Despite variation among reports, many epidemiologic studies in malnourished children reveal that early and persistent *Giardia* associates with impaired growth attainment [9, 45] despite inversely decreased stool markers of EE-like inflammation myeloperoxidase (MPO) [7] and the T-cell activation marker neopterin [9, 46]. Also, there is an apparent decreased risk for acute diarrhea and diminished markers of systemic inflammation in children infected with *Giardia* [47] that may be abolished following multi-nutrient supplementation [48]. It was critical, therefore, to examine how *Giardia* interacted alone and during co-infection. Previous findings have shown that bacteria cultivated from jejunal aspirates of patients with symptomatic giardiasis elicit more inflammation in germ free mice than axenized *Giardia* trophozoites [49], and that *Giardia* increased bacterial mucosal translocation, even after parasite clearance in some animal models [50, 51]. This led us to hypothesize that bacteria may similarly influence growth outcomes during giardiasis. Using continuous antibiotic exposures, we show for the first time that these interactions are crucial for host growth attainment. However, unlike the same antibiotic cocktail that led to reduced CD8^+^T-cell activation and consequently, decreased host-mediated immunopathogenesis in another *Giardia* model [52], we did not see significant inflammation in the mucosa of protein deficient diet fed infected mice. Therefore, the primary driver of growth impairment in this model appears to be a *Giardia*-mediated disruption in microbial-host metabolism. These data support that one mechanism of *Giardia*-mediated growth faltering is through an altered intestinal ecology [53]. For example, pipecolic acid, a byproduct of lysine degradation that is significantly increased in *Giardia* spent media [32], was uniquely detected only in *Giardia-*infected or co-infected mice, and could represent a pathway whereby intestinal parasites limit luminal availability of essential amino acids in the undernourished host [16]. Our findings of increased phosphatidylcholine (PC), choline, and taurine/isothionate in *Giardia* infected mice, may also indicate disrupted lipid metabolism through the parasite’s consumption of bile salts (independent of known expression of bile-salt hydrolases) and acquisition/turnover of exogenous lipids in the small intestine via phospholipid-transporting transmembrane proteins (such as flipases) [32], as well as choline kinases and phosphatidylcholine synthases [32]. These perturbations in bile acid and/or lipid homoestasis could have implications for growth in malnourished children [31, 54]. Finally, urinary alanine, a unique byproduct of *Giardia* glucose fermentation under low-oxygen tension [55], was elevated together with relative increases in fecal Clostridiales, suggesting an increased anaerobic environment in the *Giardia* infected mice on a protein deficient diet. Other microbial-dependent urinary metabolites that are altered during *Giardia* infection are not known to be direct products of *Giardia* metabolism: such as MA, DMA, and TMA as well as metabolites of aromatic amino acid breakdown (ie. PAG (phenylalanine), 4-CS/4-CG (tyrosine), and 3-IS/3-IAG (tryptophan)). Also, since EAEC alone also fueled microbial-dependent proteolysis of aromatic amino acids with the exception of 4-HPA sulfate, a breakdown product of tyramine that may be another unique metabolite of *Giardia* [32], we suspect these markers of amino acid catabolism indicate products of bacterial metabolism. Since decreases in the dietary constituents sucrose and tartrate in either *Giardia* or EAEC infected mice suggested reduced exogenous protein intake, this metabolic shift could have resulted from microbial degradation of host derived proteins, potentially released from injured or sloughted epithelial cells or leakage across disrupted tight-junctions. Furthermore, since these same proteolytic metabolites have been identified in undernourished children [7], and were present together with an uncoupling of TCA intermediates in Kwashiorkor-associated dysbiosis [1] our findings raise the need to elucidate the role of *Giardia* and EAEC in metabolic-based studies in human infections. Also, follow-up integrated proteomic and metabolomics analyses across various *Giardia* and EAEC strains could greatly expand the presently limited systems biology databases of these and other enteropathogens [16, 56].

Our data raise important considerations for host mucosal immune consequences of multi-enteropathogen infections in malnourished children. The lack of intestinal inflammation seen in these protein deficient diet fed mice during persistent *Giardia* infection is reminiscent of the majority of intestinal biopsies in children with *Giardia* infection [9]. This is in contrast to the persistent inflammation seen in symptomatic chronic giardiasis in adult humans [39, 57] as we previously reported in Abx-treated otherwise healthy nourished chronically infected mice [18]. Rather, our findings that *Giardia* potentiated signals of intestinal injury (mucosal IL1α and CCL11 and luminal LCN-2), but dampened markers of myeloid activation (MPO and Calprotectin) during EAEC co-infection suggests the *Giardia*-mediated mucosal immune modulation may have led to inappropriate and deleterious responses to bacterial co-infection. Notably, these findings in a persistent *G*. *lamblia* assemblage B infection model demonstrate a potentially different mechanism of *Giardia*-induced immune modulation compared to prior reports of *G*. *lamblia* assemblage A-dependent cathepsin-B mediated cleavage of IL-8 that led to reduced myeloid cell chemotactic responses described by Cotton, et al [21]. Rather, prior *Giardia* infection resulted in greater IL-4 and IL-13 and increased IL-9, a potential marker of mast cell activity [58]. The elevated CCL11 but reduced calprotectin together with an increase in Th2-type cytokines, could be consistent with the presence of intermediate-type macrophages [59] that were recently found to expand following *Giardia* challenge in mice [60]. Additional studies inclusive of more stringent flow cytometry characterization are needed to investigate the mechanisms driving this altered response. Future mechanistic studies, for example, are planned to determine whether this phenotype is the result of potential taurine-dependent inhibition of NFκB signaling in host macrophages [61].

Integrating metabolic data with altered mucosal immune responses, we identify putative microbial-mediated mechanisms driving the severity of malnutrition with cumulative pathogen exposures (Fig 5). Tryptophan, for example is required for protein synthesis and optimal host growth, but its fate is highly influenced by intestinal microbial metabolism as well as competing host tissue-compartmentalized metabolic and immune stressors. During cytokine-mediated chronic inflammation, tryptophan is metabolized in the kynurenine pathway via upregulation of indoleamine 2,3 dioxygenase (IDO) that promotes local T-cell proliferation, switching from Th17 to T- regulatory phenotypes [62] and IL-22 mediated homeostasis [63]. Alternatively, tryptophan is an important route of nicotinamide synthesis via hepatic tryptophan 2,3 dioxygenase (TDO). This production of nicotinamide increases available nicotinamide adenine dinucleotide (NAD^+^), an essential co-factor for tricarboxylic acid cycle (TCA) metabolism and preservation of oxidative phosphorylation [64]. Low serum tryptophan levels as well as increases in tryptophan degradation via IDO in the kynurenine pathway have been documented in malnourished children [3, 65], whereas urinary excretion of *N-*methly-nicotinamide (NMND) predicts catch-up growth in undernourished children [7]. In this regard, in uninfected mice fed a protein deficient diet, increased carbohydrate metabolism through the TCA cycle occurred together with increased excretion of NMND [16], suggesting increased methylation of the NAD(H) generated during the TCA cycle to NMND via the irreversible actions of nicotinamide *N*-methyltransferase (NMNT). Although NMND excretion was also increased during *Giardia* infection, the TCA cycle activity was not. Instead, during *Giardia* infection, there was an accumulation of *cis*-aconitate, a precursor to the irreversible first NAD-requiring enzyme isocitrate dehydrogenase (IDH) in the TCA cycle. This finding is compatible with an overall NAD^+^ pool deficit as a potential consequence of hypermethylation of available nicotinamide via NMNT, a potential feature of catabolic states [66]. The ability of NMNT to regulate energy expenditure [7] may ultimately be an advantageous adaptation during malnutrition. As in undernourished children [67], *Giardia-*infected mice in this model corresponded with increased ‘catch-up’ growth potential after refeeding. During EAEC infection, however, inflammatory cells likely competed for tryptophan via cytokine-mediated upregulation of IDO in the intestinal mucosa. As a result, not only was TCA activity decreased, but increased NMND was also lost during co-infection. In addition to the enhanced EAEC-mediated intestinal inflammation, when combined with *Giardia* infection, there was further evidence of microbial-mediated tryptophan breakdown. The consequences of these two intersecting pathways may have ultimately led to an insurmountable exogenous tryptophan deficit, that together with increased IL1α [68], further fueled muscle catabolism (increased creatine excretion) and exhausted cellular aerobic respiration including the loss of compensatory increases in lipid metabolism (hexanoylglycine, butyrylglycine, and isovalarylglycine and decreased acetyl-carnitine).

Although we used carefully age, sex, diet, and vendor-source matched controls to address potential biological variability when studying outcomes dependent upon intestinal microbiota, one inherent limitation of these studies performed in a specific pathogen free (SPF) environment is the inability to directly determine which microbe-microbe interactions were most consequential for pathogenesis. We speculate, therefore, that the correlation between *Enterobacteriaceae* burden and growth impairment during giardiasis and potentially enhanced EAEC virulence may be mediated through a shared *Giardia*-dependent metabolic perturbation. Recently, cultivation of a laboratory strain *E*. *coli* in *Giardia-*spent media led to conversion from a commensal to a pathogenic phenotype in a nematode infection model [69] as well as decreased taurine-receptor gene expression in the *E*. *coli* grown in *Giardia*-spent media [69]. Our *in vivo* data that *Giardia* leads to increased taurine excretion, even during EAEC co-infection, may similarly indicate decreased bacterial taurine metabolism. More rigorous co-association studies in gnotobiotic conditions are needed to differentiate *Giardia*-mediated interactions with select resident or pathogenic *Enterobacteriaceae*. Also, contrasting deleterious microbial interactions with microbes associated with health, such as *Bifidobacterium pseudolongum* [25] or *Akkermansia muciniphila* [22] that were variably reduced across groups in this model, are needed to unravel how these intriguing intestinal ecological interactions influence malnutrition.

In conclusion, this murine model of protein malnutrition and microbial disruptions using common enteropathogen challenges in malnourished children provides important insights into microbial interactions and metabolic mechanisms that contribute to undernutrition. These collective integrated studies raise important considerations for ongoing longitudinal studies of childhood malnutrition that rely upon immune and metabolic biomarkers as surrogates for small intestinal pathology. As in this model, correlating specific enteropathogen exposures with these metabolic perturbations and immune biomarkers may help to define appropriate (physiological) from maladaptive (pathological) responses, and identify critical windows of optimal and potentially individualized interventions. For example, children who excrete NMND despite enteropathogen infection, like *Giardia*-infected mice in this model, may be primed to ‘catch-up’ and thus respond to targeted nutrient therapy, whereas the lack of NMND excretion during *Giardia* infection may signify a need to identify and treat a co-existing enteropathogen such as EAEC or another trigger of intestinal inflammation. Similarly, although fecal MPO may indicate environmental enteropathy predictive of poor growth, an inappropriately suppressed MPO in the setting of an immune-modulating co-infection may be an alarm for ensuing host immune and metabolic exhaustion and morbidity. Indeed, translating integrated findings in experimental models such as those presented in this study may not only help to identify novel interventions that disrupt a vicious cycle of microbial-mediated enteric failure, but may better leverage combinations of existing interventions to restore microbial-host mutualism and promote mucosal restitution.

## Methods

### Ethics statement

This study included the use of mice. This study was conducted in strict accordance with recommendations in the Guide for the Care and Use of Laboratory Animals of the National Institutes of Health. The protocol was approved by the International Animal Care and Use Committee at the University of Virginia (Animal Care and Use Committee Protocol number: 3315). Tissue procurement was performed following anesthesia (ketamine hydrochloride and xylazine) and cervical dislocation, and all efforts were made to minimize suffering.

### Animals and malnutrition

All experiments were performed using weaned male C57Bl/6 mice received from Jackson Laboratories at 3 weeks of age. Mice were initiated on either a protein deficient diet (PD; 2% protein, Harlan Laboratories or Research Diets) or an isocaloric control diet (CD; 20% protein, Harlan Laboratories or Research Diets) within three days of arrival (24 days of life). For all experiments mice were randomized into weight-matched groups and continued on experimental diets throughout the duration of the experiment. Mice receiving continuous antimicrobials received ampicillin (1 mg/mL, Fischer), vancomycin (1 mg/mL, Novaplus), and neomycin (1.4 mg/mL, Durvet) in drinking water changed *ad libitum* or every five days. Serial weights were obtained every 1–7 days from arrival through the termination of the experiment. Stools were collected every other day following infection [18].

### *Giardia lamblia* and enteroaggregative *Escherichia coli* preparations

Gerbil-passaged purified *G*. *lamblia* H3 (Assemblage B) cysts were purchased from Waterborne, Inc. (New Orleans, LA). Cysts were washed and diluted in PBS and used within 48 hours of arrival. Each infected mouse received an inoculum of 10^4^−10^6^ cysts. *G*. *lamblia* H3 trophozoites were also obtained from Waterborne, Inc and maintained in modified TYI-S-33 media prior to inoculum preparation (10^7^/mouse) as previously described [18]. The EAEC strain 042 was originally obtained from James Nataro at the University of Virginia. For each experiment, a separate inoculum of 10^9^/mouse was grown from a glycerol stocked maintained at -80°C and prepared in DMEM high glucose medium as previously described [17]. All pathogen preparations were maintained on ice until administered via oral gavage using 22-gauged feeding needles in 100 *μ*L volumes. Uninfected controls were similarly gavaged with either 100 *μ*L of PBS (for *Giardia*) or DMEM high glucose (for EAEC) control.

### Ex vivo enumeration of *Giardia* trophozoites

At the time of euthanasia, 4-cm segments of small intestine were removed beginning 0.5 cm from the pyloric sphincter, and placed into 4-mL of chilled PBS on ice for 30 minutes. Trophozoites were identified using an inverted microscope and counted on a hemacytomer with a limit of detection of 10^4^ trophozoites/mL.

### DNA extraction for pathogen and microbiota detection

DNA from stool and/or intestinal tissue was extracted from thawed samples using the QIAmp DNA stool Kit (Qiagen) as previously described [18]. For detection of 16S rRNA genes, modifications to enrich detection including additional steps of homogenization in bead tubes (UltraClean fecal DNA bead tubes, Mo Bio Laboratories) in 400/360μL of ASL/ATL buffer using a Mini-Beadbeater for 1 minute, 30/40 μL of Proteinase K for stool/tissue homogenates, and incubation of tissues at 56°C for two hours [70].

### Real-time polymerase chain reaction for Giardia lamblia and EAEC quantification and bacterial group targets

See S1 Table for a list of gene targets used in this study. For all qPCR studies, a standard curve serial dilution for the respective target was run in replicate on all plates for validation purposes. A run was considered valid only if the BioRad CFX Detection System detected an efficiency of 90–110% and a correlation r^2^ > 0.98. Both experimental (i.e. infected animal fecal DNA) and controls (i.e. uninfected animal fecal DNA) were run on the plate. A non-template control was universally included on every plate to control for non-specific amplifications.

### *Giardia lamblia* and EAEC

Quantification of *G*. *lamblia* and EAEC infections and bacterial group targets (see bleow) were performed in a BioRad CFX Detection System by interpoloating C_t_ values of each run with a standard curve of known amounts of each respected pathogen DNA and transformed into number of organisms per milligram of sample [17, 18]. Development of these assays included spiking known quantities of pathogen into uninfected mouse stool as well as serial dilutions demonstrating no significant evidence of inhibition in the assay [17, 18]. The master mix solution and conditions for specific target detection of *G*. *lamblia* 18S small ribosomal subunit [forward primer, 5’- GACGGCTCAGGACAACGGTT-3’ (Operon), reverse primer, 5’- TTGCCAGCGGTGTCCG-3’ (Operon), and probe FAM-5’- CCCGCGGCGGTCCCTGCTAG-3’-BHQ (IDT))] and the EAEC *aap* gene (forward 5’-CTTGGGTATCAGCCTGAATG-3’ and reverse 5’-AACCCATTCGGTTAGAGCAC-3’ primers) for EAEC detection are described elsewhere [17, 18]. Amplification for *G*. *lamblia* consisted of 3 minutes at 95°C, followed by 39 cycles of 15 seconds at 95°C, and 60 seconds at 58°C. Amplification for EAEC consisted of 3min at 95°C, followed by 40 cycles of 10 seconds at 95°C and 30 seconds at 61.5°C, followed by 40 cycles of 10 seconds, starting at 65°C with 0.5°C increments for melt curve. For both pathogens, a standard curve ranging from either 10^2−^10^7^ *G*. *lamblia* cysts (determined by hemacytometer) [limit of detection 10^3^/1000 mg stool or tissue as previously described [18] or 10^1^−10^8^ EAEC CFU/mL (determined by OD_600_ and confirmed by plate counts), limit of detection 10^1^/1000 mg stool or tissue as previously described [17] was included on every PCR run. For both pathogens, sample Ct values in the range of 37–40 and/or non-specific melt curves (EAEC) were considered non-specific and excluded from the analysis.

### 16S quantification of total bacteria, firmicutes, bacteroidetes, and *Enterobacteriacea*

The SYBR Green master mix solution and conditions for detection of Firmicutes, Bacteroidetes, and Enterobacteriacea are described elsewhere [70]. The Bact934 forward primer (5’- GGARCATGTGGTTTAATTCGATGAT -3’) and Bact106 reverse primer (5’- AGCTGACGACAACCATGCAG—3’) were used for Bacteroidetes detection; Firm350 forward primer (5’—GGCAGCAGTRGGGAATCTTC—3’) and Firm814 reverse primer (5’—ACACYTAGYACTCATCGTT—3’) were used for Firmicutes detection; and Uni515 forward primer (5’—GTGCCAGCMGCCGCGGTAA—3’) and Ent826 reverse primer (5’–GCCTCAAGGGCACAACCTCCAAG—3’) for Enterobacteriaceae detection. Amplification conditions consisted of 5 minutes at 95°C, then 40 cycles of 10 seconds at 95°C and 59°C for 30 seconds. Melt curve analysis was carried out in 0.5-degree increments for 5 seconds starting at 65°C and ending with 95°C. The Ct values on each run were compared to standards of known concentrations of bacterial DNA on the same plate as previously described [71]. For universal 16S detection, we used forward primer (5’ GTGSTGCAYGGYTGTCGTCA -3’) and reverse primer (5’ ACGTCRTCCMCACCTTCCTC -3’) [72]. and an *Enterococcus faecalis* with 4x16S copies/genome as a standard. For universal 16S detection (total bacteria), SYBR Green mastermix (Biolegend) was used with 10 ng of template DNA. Amplification conditions consisted of 2 minutes at 95°C, then 45 cycles of 15 seconds at 95°C and 50°C for 30 seconds and 72°C for 45 seconds. Melt curve analysis was carried out in 0.5-degree increments for 5 seconds starting at 65°C and ending with 95°C. Any Ct values between 37–40 and/or melt curves that did not align with the respective melt curve of the known bacterial DNA standards were considered non-specific amplification and excluded from analysis.

### Total 16S V3-V4 library quantification, Illumina sequencing and data analysis

The V3-V4 region of the 16S rRNA gene was amplified according to manufacturer specifications (Ilumina Mi-Seq) from 5 ng of purified genomic DNA. Index primers (Nexterea XT Index 1 and 2) were used to label individual samples prior to library quantification. A pico-green assay was used to quantify individual sample libraries prior to normalization, pooling, and sequencing using Ilumina Mi-Seq. 16S libraries were pooled and sequenced using Ilumina MiSeq at the Microbiome Core at UNC or the Genomics Core Facility at UVA. Reads were assigned to samples using Illumina BaseSpace demultiplexing. From these reads, bacterial presence and relative abundance were quantified using the QIIME package, version 1.9.1 [73]. Fastq-join was called via QIIME to join paired-end reads with a minimum of 6 base pair overlap and 8 percent maximum difference [74]. Barcodes were extracted from paired reads, then reads were quality-filtered using split_libraries.py from QIIME with default parameters. Chimeric sequences were detected and removed using reference-based and de novo chimera identification with USEARCH61 [75] and the GreenGenes 16S rRNA database [75]. Identification of operational taxonomic units (OTUs) was performed by referencing the GreenGenes database with UCLUST (97% sequence identity cutoff) and de novo otu-picking with QIIME. The RDP classifier was used to assign taxonomy to identified OTUs. The weighted UniFrac distance [76] between each sample was calculated and principal coordinates analysis (PCoA) was performed on the resulting distance matrix. PCoA results were visualized with EMPeror [77]. To prepare OTU data for relative abundance comparisons, samples with fewer than 311 reads were excluded and OTUs were filtered by two criteria: being present in at least two samples and having 0.5% relative abundance in at least one sample. Using the remaining OTUs and their relative abundances, the DESeq2 package [78] was used, as implemented in QIIME, to determine differentially abundant OTUs. OTUs with a multiple-testing corrected p value <0.05 were considered differentially abundant.

For duodenal tissues there was a high degree of unassigned taxa. We therefore restricted the V3-V4 pipeline analysis to only those OTUs with at least 10,000 reads (“high-abundance”). This restriction resulted in 541,910 retained high quality reads in the duodenum (mean 15,483 reads/sample). 50–98% of otherwise unassigned taxa were successfully eliminated from these groups.

Metagenomic visualizations were generated using KRONA open source software (https://github.com/marbl/Krona/wiki)) after uploading OTU data files transferred to Krona ExcelTemplates (https://github.com/marbl/Krona/wiki/ExcelTemplate)) [79].

### Urine metabonomoics ^1^H NMR spectroscopy-based metabonomic analysis

Urine samples were on individual mice at each timepoint indicated. Sample collections occurred regularly at 2 pm on each collection data. Urines were placed immediately on ice, and stored at -80°C before shipping on dry ice to JMP and JS. Urines were then analyzed individually by ^1^H nuclear magnetic resonance (NMR) spectroscopy. Each sample was prepared by combining 30 μl of urine with 30 μl of phosphate buffer (pH 7.4; 100% D_2_O) containing 1 mM of the internal standard, 3-trimethylsilyl-1-[2,2,3,3-^2^H4] propionate (TSP). Samples were mixed by vortex and spun (10,000 *g*) for 10 minutes before transfer to a 1.7 mm NMR tube. Spectroscopic analysis was carried out on a 700 MHz Bruker NMR spectrometer equipped with a cryo-probe. Standard one-dimensional ^1^H NMR spectra of the urine samples were acquired with water peak suppression using a standard pulse sequence. For each sample, 8 dummy scans were followed by 128 scans and collected in 64K data points. A recycle delay of 2 s, a mixing time of 10 μs and an acquisition time of 3.8 s was used. The spectral width was set at 20 ppm. Chemical shifts in the spectra were referenced to the TSP singlet at δ 0.0. Spectra were manually phased and corrected for baseline distortions. ^1^H NMR spectra (δ 0.2–10.0) were digitized into consecutive integrated spectral regions (~20,000) of equal width (0.00055 ppm). The regions between δ 4.50–5.00 were removed in order to minimize the effect of baseline effects caused by imperfect water suppression. Each spectrum was then normalized to unit area. Multivariate modeling was performed in Matlab using in-house scripts. This included principal components analysis (PCA) using pareto scaling and orthogonal projection to latent structures-discriminant analysis (OPLS-DA) constructed using unit variance scaling. OPLS-DA models were constructed to assist model interpretation. Here, ^1^H NMR spectroscopic profiles were used as the descriptor matrix and class membership (*e*.*g*. Giardia, EAEC, uninfected) was used as the response variable. The predictive performance (Q^2^Y) of the model was calculated using a 7-fold cross validation approach and model validity was established by permutation testing (1000 permutations) [16]. Metabolites associated with a series of pair-wise OPLS-DA models were identified by the correlation coefficient (*R*) with the class membership and summarized in a heat map.

### Flow cytometry for lamina propria cells and luminex for cytokine and chemokine profiles

Flow cytometry of lamina propria cells was performed according to our previously published protocols [18]. For isolation of cells from ileum segments, suspensions of small intestinal lamina propria cells were prepared from 4 cm segments of distal small intestine beginning 1 cm from the ileocecal valve. After segments were PBS-flushed and cleaned of gross debris and mucus, they were incubated at 37°C in HBSS buffer containing 50mM EDTA and 1mM DTT for 30 minutes in a shaking incubator at 250 rpm in order to remove epithelial-layer cells. The digested tissue was passed through a 100-μm filter and the filtrate centrifuged as previously described [17]. For lamina propria cell isolations, the tissue pieces were minced and suspended in 10 ml RPMI media with 4% FBS containing 1.2 mg/ml collagenase Type IV, 1.0 mg/ml dispase, and 25–40 U/ml DNase I enzyme solution for 30 minutes at 37°C in a shaking incubator and strained through a 40-μm filter. The resulting pellets were resuspended in 1% BSA-PBS buffer. Fluorophore-conjugated purified mAbs used in flow cytometry were purchased from BD Biosciences (CD4-PE-Cy7, CD3ε-BV421, CD45-V500, and CD11b-APC-Cy7 (clone M1/70)) and Biolegend (B220 [CD45R]-PerCP), and cell surface staining was performed according to the manufacturer’s instructions. All samples were acquired on a CyAn ADP LX analyzer (BD Biosciences and Cytek Development). The leukocyte population was gated based on forward/side scatter, the threshold was set at 50 FSC and single cells isolated via pulse width. All gates were applied universally to all samples within each batch. Data is represented as number positive per normalized total events (i.e., 500,000) or frequency within a specified gate. This methodology was used to elucidate the proportional leukocyte changes in equal amounts of tissue [80]. Cell analysis was performed using FlowJo version 9.3.3 software (Tree Star).

For mucosal cytokine and chemokine responses, 0.5–1.0 cm of ileum were immediately placed in liquid nitrogen at the time of euthanasia and stored at -80°C until use. Protein was collected from ileum lysates, which were made using a lysis buffer containing 50 mM HEPES, 1% Triton X-100, and Halt protease inhibitor on ice and homogenized in Zirconia beads (Biospec) using a Mini-Beadbeater (Biospec) for 60 seconds. Clarified supernatants were stored at -80°C. Multiplex protein quantification was performed using Luminex 100 IS System at the University of Virginia Biomolecular Core facility.

### Cecal/Fecal inflammatory marker measurement

Markers of inflammation were measured in the cecal contents or stool using methods described previously [5, 16]. Briefly, stool or cecal contents were collected when cecal and other intestinal tissue was harvested and stored at -20°C until measurement. At the time of analysis specimens were allowed to thaw at room temperature and were diluted 7-fold in buffer with protease inhibitors (RIPA, radioimmunoprecipitation assay buffer). The samples were vortexed, centrifuged and the supernatants used to measure the biomarkers. The fecal myeloperoxidase (MPO) and lipocalin-2 concentrations were measured using commercially available kits that employed polyclonal antibody-based enzyme-linked immunosorbent assay (ELISA) methods, from R&D systems (Minneapolis, USA) according the manufacturer’s instructions. Cecal calprotectin was quantified by ELISA (Hycult Biotech) according to manufacturer's instructions using 1:1000 dilution of cecal contents based on optimization using pooled cecal samples. Measured protein levels were normalized to total lysate protein for respective specimens as determined by bicinchoninic assay (BCA) (Thermoscientific) at 562nm absorbance (Biotek ELISA plate reader) after 30 minutes incubation of sample with reagent at room temperature. Total protein of each sample was assayed using the BCA Protein Assay Kit from Pierce (Pittsburgh, PA). The absorption was measured using an Epoch plate reader, Bio-tek Instruments, Inc. (USA). Units were expressed as pg/mg of total protein.

### Statistical analysis

Data analyses were performed with GraphPad Prism 6.0 and 7.0 software. All statistical analyses were done from raw data with the use of One-way and Two-way analysis of variance with Dunn’s or Bonferroni post hoc analysis from multi-group comparisons. For comparisons between only two groups, Student *t* tests were used for parametrically distributed data and Mann-Whitney tests for non-parametrically distributed data where applicable. Differences were considered significant at *P*<0.05. Data are represented as means ± standard errors of the mean.

## Supporting information

S1 Fig A) Small intestinal *Giardia* burden in mice fed CD 35 days after 10^6^ *G*. *lamblia* H3 cyst challenge. Mice received Abx beginning 8 days prior to challenge and through 21 days post-challenge. B) *Giardia* stool shedding (28 through 42 days post-infection) by 18S small ribosomal subunit qPCR in weaned mice fed control diet (CD) (left) or protein deficient diet (PD) (right) receiving continuous antibiotics (vancomycin, neomycin, and ampicillin) in drinking water beginning 8 days prior to 10^6^ *G*. *lamblia* H3 cyst challenge. C) Comparison of *G*. *lamblia* H3 colonization in duodenum 7 days following challenge with either axenized trophozoites (10^7^/100 mcl) or gerbil-passaged purified cysts (10^6^/100 mcl) in mice fed PD with or without antibiotics as determined by light microscopy (left) and in a subset comparing quantification by light microscopy and qPCR (right). * = P<0.05 as indicated; *** = P<0.001 for % infectivity trophozoites (33%; 1/3) vs cysts (92%; 12/13). D) Ratio of Firmicutes:Bacteroidetes (F:B) in small intestine of mice fed either CD or PD after 20 days on respective diets. (*P<0.05, n = 6–7 per group). E) Impact of continuous antibiotics on fecal (left) and duodenal (right) 16S V3-V4 community taxa in mice fed PD diet with and without *Giardia* infection through 15 days post-challenge. Only high abundance (>10,000 reads/OTU) taxa are shown due to high-proportion low-abundances of unassigned taxa in antibiotic-treated animals (see Methods). *P<0.05, ****P<0.0001 for comparisons as indicated.
(PDF)

S2 Fig *Giardia* increases neither small intestinal mucosal leukocyte numbers nor pro-inflammatory cytokine responses in protein malnourished mice. (A) Flow cytometry of ileum lamina propria leukocytes (LPL) day 17 post-challenge in PD-fed mice. (B) Representative chemokine and cytokine proteins (by Luminex) secreted in ileum of mice fed PD. n = 6-12/group, *P<0.05 as indicated.
(PDF)

S3 Fig A) Effect of *Giardia* (d7 (row a) and d13 post-challenge (row b)), EAEC (d7 post-challenge (row c)), or both (rows d-f) on 16S V3-V4 fecal bacterial community during protein deficiency. Sub-phyla level OTU relative abundances as indicated. *P<0.05 for genus-level abundances highlighted in green (increased in experimental group, leftmost) or red (decreased in experimental group, leftmost) in the table. OTUs included in statistical tests were filtered by two criteria: presence in at least two samples in the dataset, and total relative abundance across all samples > 0.5%. B-D) PCA score plots (left) and OPLS-DA correlation coefficient plots (right) indicating the differences between: B) Mice at day 7 post infection with EAEC (experimental day 13 in Fig 4) versus their corresponding age and protein deficient diet fed-matched uninfected controls; C) co-infected mice (*Giardia* d13, EAEC d7) versus age and diet-matched uninfected controls; D) Mice infected with *Giardia* (d13) versus age and diet-matched co-infected mice. E-G) *Giardia* facilitates catch-up growth in mice fed a protein deficient diet. E) Growth in mice fed protein-deficient diet (PD) through 42 days after *G*. *lamblia* H3 cyst (10^6^) infection. F) Growth as % initial weight after transitioning mice from PD to control diet (day 0 on x-axis) and through 25 days post-refeeding (*P<0.05). C) Persistence of *Giardia* in feces and small intestine after re-feeding (D42 = day of re-feeding). (n = 2-4/group).
(PDF)

S1 Table List of gene targets and primer sequences used in this study. (PPTX)
