# Supplementary figures and images for: Cross-modulation of pathogen-specific pathways enhances malnutrition during enteric co-infection with Giardia lamblia and enteroaggregative Escherichia coli
Source: PLoS Pathog. 2017 Jul 27;13(7):e1006471. doi: 10.1371/journal.ppat.1006471 (PMC5549954; doi:10.1371/journal.ppat.1006471)

**A**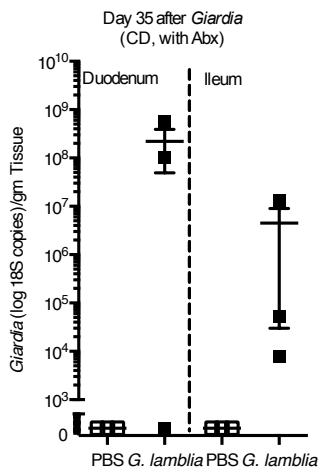**B**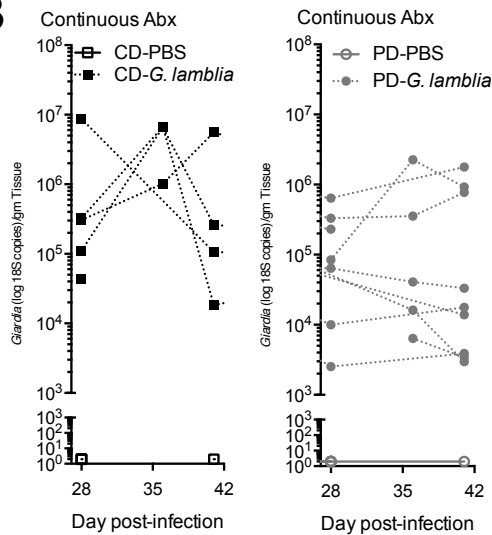**C**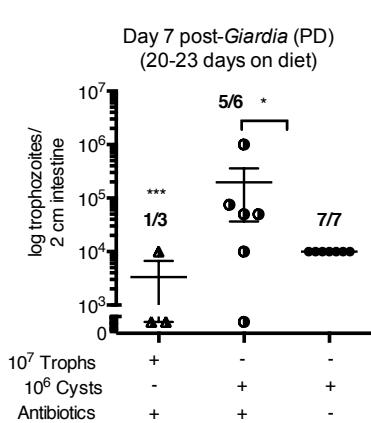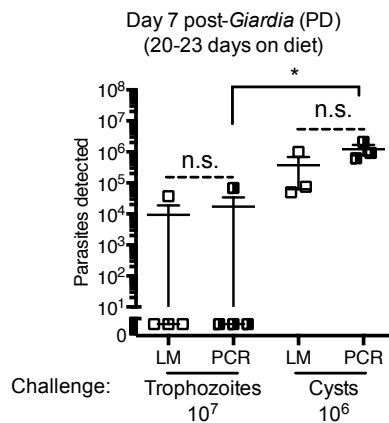**D**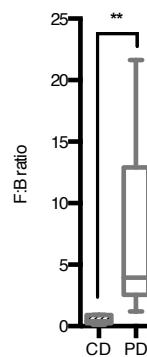**E**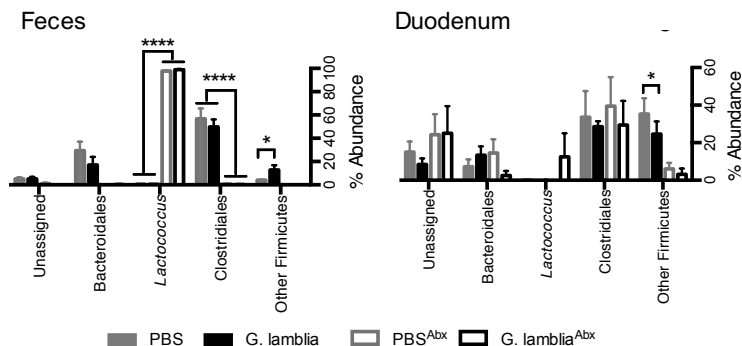

Supplement: S1 Fig — A) Small intestinal Giardia burden in mice fed CD 35 days after 106 G. lamblia H3 cyst challenge. Mice received Abx beginning 8 days prior to challenge and through 21 days post-challenge. B) Giardia stool shedding (28 through 42 days post-infection) by 18S small ribosomal subunit qPCR in weaned mice fed control diet (CD) (left) or protein deficient diet (PD) (right) receiving continuous antibiotics (vancomycin, neomycin, and ampicillin) in drinking water beginning 8 days prior to 106 G. lamblia H3 cyst challenge. C) Comparison of G. lamblia H3 colonization in duodenum 7 days following challenge with either axenized trophozoites (107/100 mcl) or gerbil-passaged purified cysts (106/100 mcl) in mice fed PD with or without antibiotics as determined by light microscopy (left) and in a subset comparing quantification by light microscopy and qPCR (right). * = P<0.05 as indicated; *** = P<0.001 for % infectivity trophozoites (33%; 1/3) vs cysts (92%; 12/13). D) Ratio of Firmicutes:Bacteroidetes (F:B) in small intestine of mice fed either CD or PD after 20 days on respective diets. (*P<0.05, n = 6–7 per group). E) Impact of continuous antibiotics on fecal (left) and duodenal (right) 16S V3-V4 community taxa in mice fed PD diet with and without Giardia infection through 15 days post-challenge. Only high abundance (>10,000 reads/OTU) taxa are shown due to high-proportion low-abundances of unassigned taxa in antibiotic-treated animals (see Methods). *P<0.05, ****P<0.0001 for comparisons as indicated. (PDF) [file ppat.1006471.s001.pdf]

Day 17 post-*Giardia* (32 days on diet)

**A**

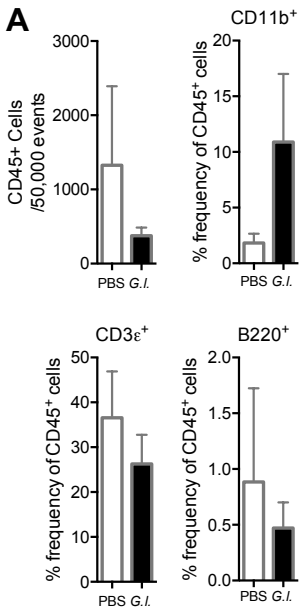

**B**

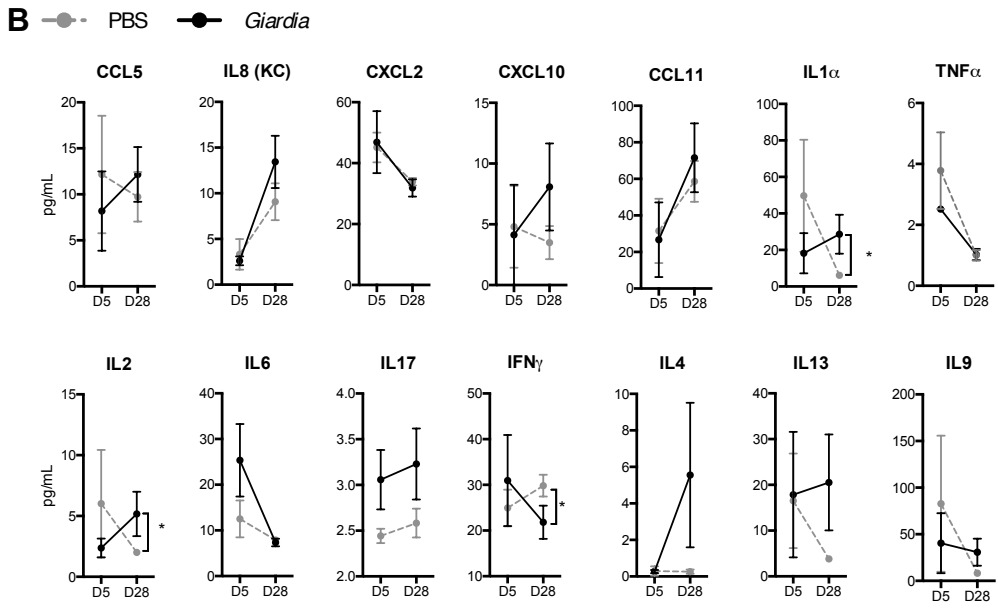

Supplement: S2 Fig — (A) Flow cytometry of ileum lamina propria leukocytes (LPL) day 17 post-challenge in PD-fed mice. (B) Representative chemokine and cytokine proteins (by Luminex) secreted in ileum of mice fed PD. n = 6-12/group, *P<0.05 as indicated. (PDF) [file ppat.1006471.s002.pdf]
