# Supplementary material for: Cross-modulation of pathogen-specific pathways enhances malnutrition during enteric co-infection with Giardia lamblia and enteroaggregative Escherichia coli
Source: PLoS Pathog. 2017 Jul 27;13(7):e1006471. doi: 10.1371/journal.ppat.1006471 (PMC5549954; doi:10.1371/journal.ppat.1006471)

A

	Bacteroidetes	Firmicutes; c_Bacilli		Firmicutes; c_Clostridia				Verrucomicrobia	Actinobacteria	Proteobacteria
Groups	o_Bacteroidiales; f_S24-7 Unnamed	*Turicibacter sp.*	f_Enteroccaceae Unnamed	o_Clostridiales Unnamed	f_Clostridiaceae Unnamed	f_Ruminococcaceae Unnamed	*Oscillospira sp.*	*Akkermansia muciniphila*	*Bifidobacterium pseudolongum*	Enterobacteriaceae Unnamed
a Giardia d7 vs PBS d7	-1.758418625	2.759836878		3.163012639	1.165082123	-1.592197822		-7.367051691		
b Giardia d13 vs PBS d13	1.021401154		3.70665638		2.632456946			-5.931232957	-3.111689357	
c EAEC vs PBS d13		2.454798862							-3.766448412	7.154464932
d Giardia-EAEC vs PBS d13						2.741721101				5.146253263
e Giardia-EAEC vs Giardia d13								6.011953179		4.516694352
f Giardia-EAEC vs EAEC						2.175999124				

B **EAEC alone**

OPLS-DA coefficients plot ($Q^2Y = 0.55$; $p = 0.022$)

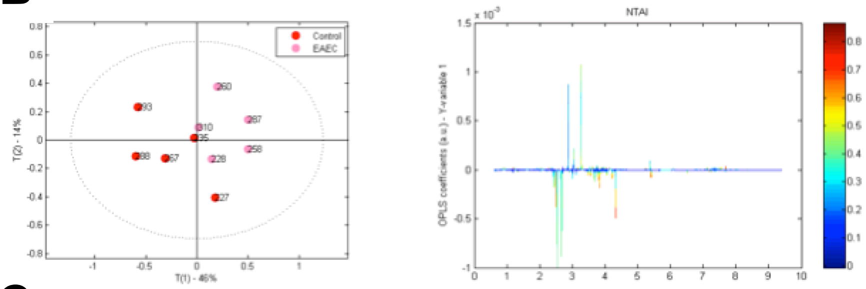

C **(Giardia + EAEC) vs Control**

OPLS-DA coefficients plot ($Q^2Y = 0.61$; $p = 0.014$)

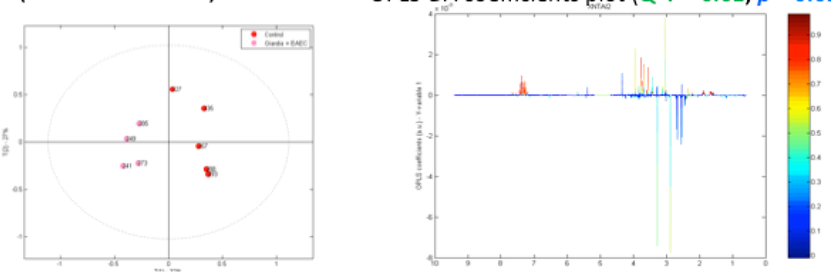

D **Giardia vs (EAEC + Giardia)**

OPLS-DA coefficients plot ($Q^2Y = 0.48$; $p = 0.040$)

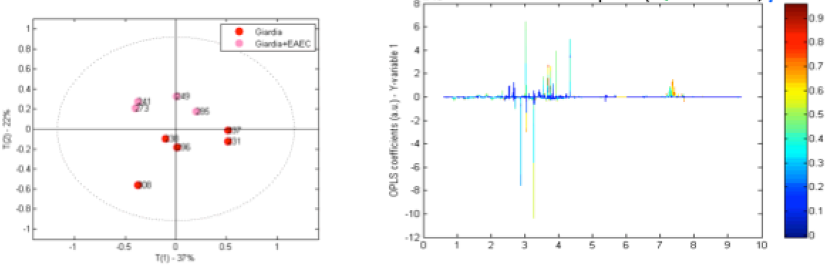

E

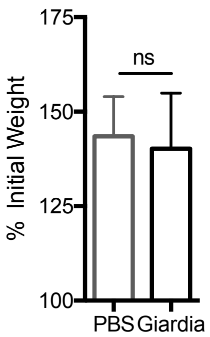

F

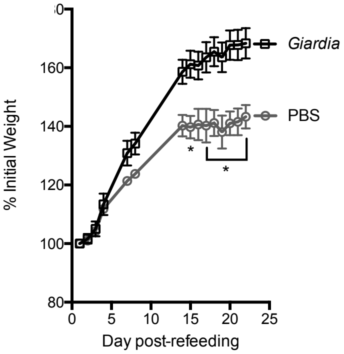

G

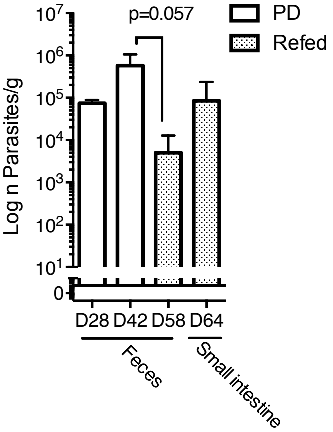

Supplement: S3 Fig — A) Effect of Giardia (d7 (row a) and d13 post-challenge (row b)), EAEC (d7 post-challenge (row c)), or both (rows d-f) on 16S V3-V4 fecal bacterial community during protein deficiency. Sub-phyla level OTU relative abundances as indicated. *P<0.05 for genus-level abundances highlighted in green (increased in experimental group, leftmost) or red (decreased in experimental group, leftmost) in the table. OTUs included in statistical tests were filtered by two criteria: presence in at least two samples in the dataset, and total relative abundance across all samples > 0.5%. B-D) PCA score plots (left) and OPLS-DA correlation coefficient plots (right) indicating the differences between: B) Mice at day 7 post infection with EAEC (experimental day 13 in Fig 4) versus their corresponding age and protein deficient diet fed-matched uninfected controls; C) co-infected mice (Giardia d13, EAEC d7) versus age and diet-matched uninfected controls; D) Mice infected with Giardia (d13) versus age and diet-matched co-infected mice. E-G) Giardia facilitates catch-up growth in mice fed a protein deficient diet. E) Growth in mice fed protein-deficient diet (PD) through 42 days after G. lamblia H3 cyst (106) infection. F) Growth as % initial weight after transitioning mice from PD to control diet (day 0 on x-axis) and through 25 days post-refeeding (*P<0.05). C) Persistence of Giardia in feces and small intestine after re-feeding (D42 = day of re-feeding). (n = 2-4/group). (PDF) [file ppat.1006471.s003.pdf]
