# Supplementary material for: Cross-modulation of pathogen-specific pathways enhances malnutrition during enteric co-infection with Giardia lamblia and enteroaggregative Escherichia coli
Source: PLoS Pathog. 2017 Jul 27;13(7):e1006471. doi: 10.1371/journal.ppat.1006471 (PMC5549954; doi:10.1371/journal.ppat.1006471)
Supplement: S1 Table — (PPTX) [file ppat.1006471.s004.pptx]

## Slide 1
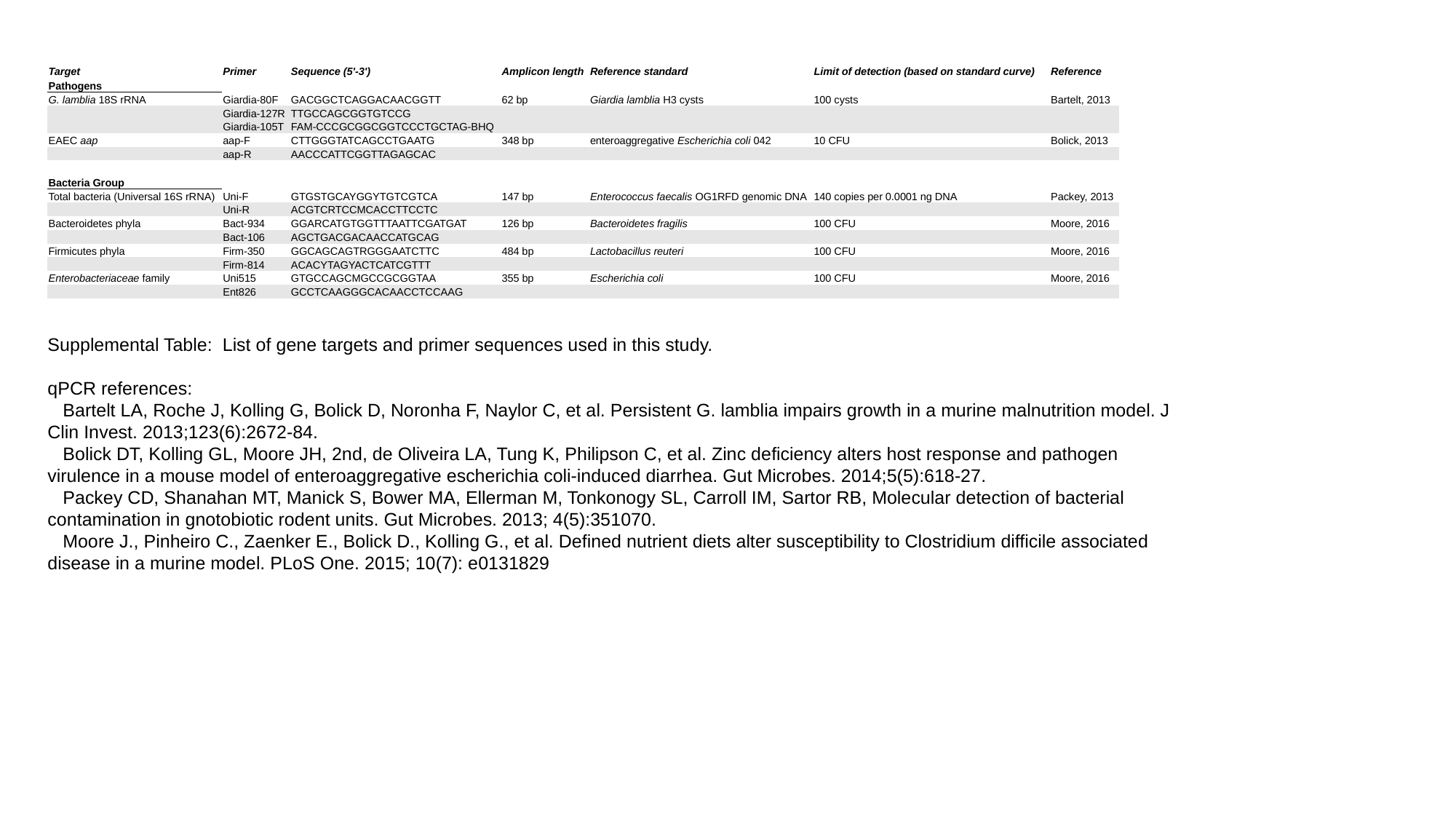

| Target | Primer | Sequence (5'-3') | Amplicon length | Reference standard | Limit of detection (based on standard curve) | Reference |
| --- | --- | --- | --- | --- | --- | --- |
| Pathogens | | | | | | |
| G. lamblia 18S rRNA | Giardia-80F | GACGGCTCAGGACAACGGTT | 62 bp | Giardia lamblia H3 cysts | 100 cysts | Bartelt, 2013 |
| | Giardia-127R | TTGCCAGCGGTGTCCG | | | | |
| | Giardia-105T | FAM-CCCGCGGCGGTCCCTGCTAG-BHQ | | | | |
| EAEC aap | aap-F | CTTGGGTATCAGCCTGAATG | 348 bp | enteroaggregative Escherichia coli 042 | 10 CFU | Bolick, 2013 |
| | aap-R | AACCCATTCGGTTAGAGCAC | | | | |
| | | | | | | |
| Bacteria Group | | | | | | |
| Total bacteria (Universal 16S rRNA) | Uni-F | GTGSTGCAYGGYTGTCGTCA | 147 bp | Enterococcus faecalis OG1RFD genomic DNA | 140 copies per 0.0001 ng DNA | Packey, 2013 |
| | Uni-R | ACGTCRTCCMCACCTTCCTC | | | | |
| Bacteroidetes phyla | Bact-934 | GGARCATGTGGTTTAATTCGATGAT | 126 bp | Bacteroidetes fragilis | 100 CFU | Moore, 2016 |
| | Bact-106 | AGCTGACGACAACCATGCAG | | | | |
| Firmicutes phyla | Firm-350 | GGCAGCAGTRGGGAATCTTC | 484 bp | Lactobacillus reuteri | 100 CFU | Moore, 2016 |
| | Firm-814 | ACACYTAGYACTCATCGTTT | | | | |
| Enterobacteriaceae family | Uni515 | GTGCCAGCMGCCGCGGTAA | 355 bp | Escherichia coli | 100 CFU | Moore, 2016 |
| | Ent826 | GCCTCAAGGGCACAACCTCCAAG | | | | |
Supplemental Table: List of gene targets and primer sequences used in this study.
qPCR references:
 Bartelt LA, Roche J, Kolling G, Bolick D, Noronha F, Naylor C, et al. Persistent G. lamblia impairs growth in a murine malnutrition model. J Clin Invest. 2013;123(6):2672-84.
 Bolick DT, Kolling GL, Moore JH, 2nd, de Oliveira LA, Tung K, Philipson C, et al. Zinc deficiency alters host response and pathogen virulence in a mouse model of enteroaggregative escherichia coli-induced diarrhea. Gut Microbes. 2014;5(5):618-27.
 Packey CD, Shanahan MT, Manick S, Bower MA, Ellerman M, Tonkonogy SL, Carroll IM, Sartor RB, Molecular detection of bacterial contamination in gnotobiotic rodent units. Gut Microbes. 2013; 4(5):351070.
 Moore J., Pinheiro C., Zaenker E., Bolick D., Kolling G., et al. Defined nutrient diets alter susceptibility to Clostridium difficile associated disease in a murine model. PLoS One. 2015; 10(7): e0131829
